# Inbreeding depression across the genome of Dutch Holstein Friesian dairy cattle

**DOI:** 10.1186/s12711-020-00583-1

**Published:** 2020-10-28

**Authors:** Harmen P. Doekes, Piter Bijma, Roel F. Veerkamp, Gerben de Jong, Yvonne C. J. Wientjes, Jack J. Windig

**Affiliations:** 1 Animal Breeding and Genomics, Wageningen University and Research, P.O. Box 338, 6700 AH Wageningen, The Netherlands; 2 Centre for Genetic Resources the Netherlands, Wageningen University and Research, P.O. Box 338, 6700 AH Wageningen, The Netherlands; 3 Cooperation CRV, Wassenaarweg 20, 6843 NW Arnhem, The Netherlands

## Abstract

**Background:**

Inbreeding depression refers to the decrease in mean performance due to inbreeding. Inbreeding depression is caused by an increase in homozygosity and reduced expression of (on average) favourable dominance effects. Dominance effects and allele frequencies differ across loci, and consequently inbreeding depression is expected to differ along the genome. In this study, we investigated differences in inbreeding depression across the genome of Dutch Holstein Friesian cattle, by estimating dominance effects and effects of regions of homozygosity (ROH).

**Methods:**

Genotype (75 k) and phenotype data of 38,792 cows were used. For nine yield, fertility and udder health traits, GREML models were run to estimate genome-wide inbreeding depression and estimate additive, dominance and ROH variance components. For this purpose, we introduced a ROH-based relationship matrix. Additive, dominance and ROH effects per SNP were obtained through back-solving. In addition, a single SNP GWAS was performed to identify significant additive, dominance or ROH associations.

**Results:**

Genome-wide inbreeding depression was observed for all yield, fertility and udder health traits. For example, a 1% increase in genome-wide homozygosity was associated with a decrease in 305-d milk yield of approximately 99 kg. For yield traits only, including dominance and ROH effects in the GREML model resulted in a better fit (*P* < 0.05) than a model with only additive effects. After correcting for the effect of genome-wide homozygosity, dominance and ROH variance explained less than 1% of the phenotypic variance for all traits. Furthermore, dominance and ROH effects were distributed evenly along the genome. The most notable region with a favourable dominance effect for yield traits was on chromosome 5, but overall few regions with large favourable dominance effects and significant dominance associations were detected. No significant ROH-associations were found.

**Conclusions:**

Inbreeding depression was distributed quite equally along the genome and was well captured by genome-wide homozygosity. These findings suggest that, based on 75 k SNP data, there is little benefit of accounting for region-specific inbreeding depression in selection schemes.

## Background

Inbreeding depression refers to the decrease in mean performance with increased levels of inbreeding [1]. Many important traits in dairy cattle show inbreeding depression [2–5]. For example, a 1% increase in pedigree-based inbreeding is associated with a decrease in 305-day milk yield of 20 to 38 kg and with an increase in calving interval of 0.2 to 0.7 days [6–8]. The reduction in mean performance is believed to be caused by the increase in homozygosity associated with inbreeding, reducing the expression of dominance effects [1, 9]. When dominance effects are on average favourable (i.e. when there is directional dominance in the favourable direction), their reduced expression results in a lower phenotypic performance. Not all genomic loci are expected to contribute equally to inbreeding depression. The expected contribution of a locus depends on both its dominance effect (higher with larger dominance effect) and its allele frequency (higher at intermediate allele frequencies) [1, 9]. Interactions between loci, i.e. epistasis, may play a role in explaining inbreeding depression as well. However, epistasis is difficult to prove and difficult to account for in statistical models. Therefore, epistasis is typically ignored. When epistasis is ignored, the change in mean phenotypic performance due to inbreeding equals $-F{\sum }_{i}{2p}_{i}{q}_{i}{d}_{i}$, where $F$ is the genome-wide inbreeding coefficient, ${d}_{i}$ is the statistical dominance effect at locus $i$, and ${p}_{i}$ and ${q}_{i}$ are the allelic frequencies [1].

The increasing availability of single nucleotide polymorphism (SNP) data enables the study of inbreeding depression along the genome. SNPs are expected to capture effects of quantitative trait loci (QTL) in linkage disequilibrium (LD) with the SNPs. Traditionally, single SNP genome-wide association studies (GWAS) have been conducted to identify significant dominance (and additive) associations [10–12]. In such studies, one SNP is fitted at a time and typically a pedigree-based or genomic relationship matrix is included to account for population structure and prevent inflation of type I errors (e.g. [12]). With approaches that are more novel, all SNP effects can be estimated simultaneously. For example, additive genetic and dominance relationship matrices can be computed [13] and these matrices can be fitted in a mixed model using genomic best linear unbiased prediction (GBLUP) to estimate additive genetic and dominance effects, after which additive and dominance effects of single SNPs can be obtained through back-solving (e.g. [14]). Since variance components need to be estimated, genomic residual maximum likelihood (GREML) can be used, based on the same mixed model, yielding estimates of random effects and variance components simultaneously, whereas GBLUP assumes that variances are known [15]. Benefits of GREML SNP solutions, over those from a single SNP GWAS, are that all SNP effects are estimated simultaneously (i.e. accounting for other SNPs in LD) and that effects are regressed towards the mean depending on information in the data.

In addition to the estimation of dominance effects, there is an increasing interest in the use of regions of homozygosity (ROH) to quantify inbreeding and inbreeding depression [2, 4, 9, 16]. When compared to homozygosity of individual SNPs, ROH may better capture (region-specific differences in) inbreeding depression. This is due to two reasons. First, as a multi-locus measure of LD, ROH are expected to better capture the probability that QTL located in between the SNPs of the ROH are homozygous. When many subsequent SNPs are homozygous, i.e. are in ROH, it is very likely that the loci between those SNPs are also homozygous. Homozygosity of individual SNPs is expected to be less predictive of the homozygosity at QTL, because of the strong dependence on the LD between the QTL and the individual SNPs (e.g. [45]). Second, ROH capture more recent inbreeding, and recent inbreeding is expected to be more harmful than old inbreeding, although empirical results do not always support this hypothesis [2, 8]. In a simulation study, Keller et al. [17] found that, among the inbreeding measures that they investigated, ROH-based inbreeding performed best in capturing the homozygous inbreeding load. Martikainen et al. [4] estimated the effect of ROH-based inbreeding on fertility traits in Finnish Ayrshire cattle, first per chromosome and then within chromosomes using a sliding window approach. Pryce et al. [2] performed a single SNP GWAS to study the effect of ROH on yield traits and calving interval in Australian Holstein and Jersey cattle. In their approach, the ROH-status of a SNP was set to 1 when the SNP was in a ROH (irrespective of which ROH), and to 0 otherwise [2]. Ferenčaković et al. [16] performed a similar analysis for sperm quality traits in Austrian Fleckvieh bulls. Although these studies did report candidate regions associated with inbreeding depression, they did not consider how much of the total phenotypic variation was explained by ROH effects (in relation to additive and dominance effects).

The objective of this study was to estimate dominance and ROH effects across the genome for Dutch Holstein Friesian dairy cattle and to estimate the contribution of these effects to the phenotypic variance. For various yield, fertility and udder health traits, first we ran GREML models to estimate the effect of genome-wide homozygosity and estimate the amount of variance attributable to additive, dominance and ROH effects. Then, we obtained individual SNP effects through back-solving. We also performed a single SNP GWAS to estimate additive, dominance and ROH effects per SNP and compared GWAS estimates with those obtained from the GREML approach.

## Methods

### Data

In total, 38,792 first-parity cows (fraction Holstein Friesian > 87.5%, either red or black), which calved in the period 2012–2016 in 233 herds, were included. The same dataset was used as in Doekes et al. [8]. Genotype and phenotype data were provided by the Dutch-Flemish cattle improvement co-operative (CRV; Arnhem, the Netherlands). Cows were genotyped with the Illumina BovineSNP50 BeadChip (v1 and v2) or CRV custom-made 60 k Illumina panel (v1 and v2). Genotypes were imputed to approximately 76 k, following Druet et al. [18]. The 75,538 SNPs used by Doekes et al. [8] were remapped to the ARS-UCD1.2 assembly, using the NAGRP Data Repository [19] and the NCBI Genome Remapping Service [20]. The final dataset comprised 75,377 successfully remapped SNPs. The distribution of the allelic frequencies of these SNPs was approximately uniform [see Additional file 1: Figure S1].

Phenotypic data included yield, fertility and udder health traits. For yield, the 305-day milk yield (MY; in kg), 305-day fat yield (FY; in kg) and 305-day protein yield (PY; in kg) were included. For fertility, the calving interval (CI; in days), interval calving to first insemination (ICF; in days), interval first to last insemination (IFL; in days) and conception rate (CR; in %) were included. For udder health, the mean somatic cell scores for day 5 through to 150 (SCS150; in units) and day 151 through to 400 (SCS400; in units) were included. Somatic cell scores were calculated as 1000 + 100*[log2 of cells/mL]. Descriptive statistics for the different traits are reported by Doekes et al. [8].

### Identification of ROH

Regions of homozygosity (ROH) were identified with the Plink 2.0 software [21]. The following criteria were used to define a ROH: (i) a minimum physical length of 1 Mb, (ii) a minimum of 15 SNPs, (iii) a minimum density of 1 SNP per 100 kb, (iv) a maximum of 1 heterozygous call within a ROH, and (v) a maximum gap of 500 kb between two consecutive SNPs. Since genotypes were imputed to 76 k, there were no missing genotypes. A scanning window of 15 SNPs was used, with a maximum of 1 heterozygote call per window. The Plink command was *plink --cow --homozyg --homozyg-density 100 --homozyg-gap 500 --homozyg-het 1 --homozyg-kb 1000 --homozyg-snp 15 --homozyg-window-het 1 --homozyg-window-snp 15*. The use of criteria such as a maximum gap of 500 kb will have resulted in some SNPs having a lower probability of being in a ROH (e.g. there were 66 gaps of > 500 kb), but will also have reduced the number of false positive ROH. The SNP density was relatively uniform along the genome (see Additional file 1: of Doekes et al. [22]).

### Statistical models

Additive, dominance and ROH effects were estimated with two approaches: (i) a GREML model with back-solving, and (ii) a single SNP GWAS. For both approaches, the classical (“statistical”) parametrization was used for additive and dominance effects, which implies among others that additive effects were calculated as allele substitution effects (see Vitezica et al. [13]).

### GREML with back-solving

GREML models were used to estimate all SNP effects simultaneously and to estimate variance components. For each trait, three models were run in mtg2 [23]: one with only additive effects (A), one with additive and dominance effects (AD), and one with additive, dominance and ROH effects (ADR). Model A was:

$${\mathbf{y}} = {\mathbf{Xb}} + {\mathbf{Qc}} + {\mathbf{u}} + {\mathbf{e}}, \quad \quad \quad {\text{Model A}}$$

where $\mathbf{y}$ is a vector of phenotypes; $\mathbf{X}$ is an incidence matrix that related the observations to fixed effects; $\mathbf{b}$ is a vector of fixed effects that included herd of calving (233 levels), year of calving (5 levels), season of calving (4 levels, defined as the four quarters of a year), age at calving (as linear covariate) and genome-wide SNP homozygosity (as linear covariate, to account for genome-wide inbreeding depression), the latter being calculated as the proportion of homozygous SNPs; $\mathbf{Q}$ is an incidence matrix that related observations to random herd-year-season effects; $\mathbf{c}$ is a vector of random herd-year-season effects (4596 levels), which were assumed to be distributed as $\mathbf{c}\sim N(\mathbf{0},\mathbf{I}{\sigma }_{HYS}^{2})$, with ${\sigma }_{HYS}^{2}$ being the herd-year-season variance and $\mathbf{I}$ an identity matrix; $\mathbf{u}$ is a vector of random polygenic additive effects (i.e. breeding values), which were assumed to be distributed as $\mathbf{u}\sim N(\mathbf{0},\mathbf{G}{\sigma }_{A}^{2})$, with $\mathbf{G}$ being the genomic relationship matrix and ${\sigma }_{A}^{2}$ the additive genetic variance; and $\mathbf{e}$ is a vector of random residuals, which were assumed to be distributed as $\mathbf{e}\sim N(\mathbf{0},\mathbf{I}{\sigma }_{E}^{2})$, with $\mathbf{I}$ being an identity matrix and ${\sigma }_{E}^{2}$ the residual variance.

Model A was extended to model AD by adding a dominance term:

$${\mathbf{y}} = {\mathbf{Xb}} + {\mathbf{Qc}} + {\mathbf{u}} + {\mathbf{v}} + {\mathbf{e}},\quad \quad \quad {\text{Model AD}}$$

where $\mathbf{v}$ is a vector of random polygenic dominance deviations, which were assumed to be distributed as $\mathbf{v}\sim N(\mathbf{0},\mathbf{D}{\sigma }_{D}^{2})$, with $\mathbf{D}$ being the dominance relationship matrix and ${\sigma }_{D}^{2}$ the dominance variance.

Model AD was further extended to model ADR by adding a ROH term:

$${\mathbf{y}} = {\mathbf{Xb}} + {\mathbf{Qc}} + {\mathbf{u}} + {\mathbf{v}} + {\mathbf{w}} + {\mathbf{e}},\quad \quad \quad {\text{Model ADR}}$$

where $\mathbf{w}$ is a vector of random polygenic ROH deviations, which were assumed to be distributed as $\mathbf{w}\sim N(\mathbf{0},\mathbf{R}{\sigma }_{ROH}^{2})$, with $\mathbf{R}$ being a ROH-based relationship matrix and ${\sigma }_{ROH}^{2}$ the ROH variance.

The additive genomic relationship matrix ($\mathbf{G}$) was computed with *calc_grm* [24], according to VanRaden [25]:

$$\mathbf{G}=\frac{\mathbf{Z}{\mathbf{Z}}^{\mathbf{^{\prime}}}}{{\sum }_{i}2{p}_{i}{q}_{i}}$$

where ${p}_{i}$ is the allele frequency of allele *A* at SNP $i$, ${q}_{i}$ the allele frequency of allele *B* at SNP $i$ and $\mathbf{Z}$ is the additive marker covariate matrix with elements of $-2{p}_{i}$, $1-2{p}_{i}$, and $2-2{p}_{i}$ for genotypes *BB*, *AB*, and *AA*, respectively.

The dominance relationship matrix ($\mathbf{D}$) was computed with *calc_grm* [24], according to Vitezica et al. [13]:

$$\mathbf{D}=\frac{{\mathbf{H}\mathbf{H}}^{\mathbf{^{\prime}}}}{{\sum }_{i}{\left(2{p}_{i}{q}_{i}\right)}^{2}},$$

where $\mathbf{H}$ is the dominance marker covariate matrix with elements of$-2{{p}_{i}}^{2}$, $2{p}_{i}{q}_{i}$, $-2{{q}_{i}}^{2}$ for genotypes *BB*, *AB*, and *AA*, respectively.

The ROH-based relationship matrix ($\mathbf{R}$) was introduced in this study to quantify the effect of a SNP being in a ROH (irrespective of which ROH). $\mathbf{R}$ was computed as a cross-product matrix:

$$\mathbf{R}=\frac{\mathbf{M}{\mathbf{M}}^{\mathbf{^{\prime}}}}{{\sum }_{i}{p}_{i}^{*}{q}_{i}^{*}},$$

where ${p}_{i}^{*}$ is the frequency of SNP $i$ being in a ROH, ${q}_{i}^{*}$ is the frequency of SNP $i$ not being in a ROH, and $\mathbf{M}$ is the ROH marker covariate matrix with elements of $1-{p}_{i}^{*}$ for being in a ROH and of $0-{p}_{i}^{*}$ for not being in ROH. Hence, element ${R}_{jk}$ could be interpreted as the (centered and scaled) probability that animals $j$ and $k$ both have a ROH at a random SNP. Note that the ROH does not have to be the exact same ROH for both animals. For example, if animal $j$ has a ROH ranging from the 10th to the 40th SNP, and animal $k$ has a ROH ranging from the 20th to the 45th SNP, then both animals “share” a ROH from the 20th SNP to the 40th SNP. Even if animals “share” a ROH at the same location, the ROH does not have to be identical. Namely, animal $j$ could have a ROH with allele 1, and animal $k$ with allele 2. It is important to note that the $\mathbf{R}$ matrix is different from the segment-based (or haplotype-based) relationship matrix that was defined by De Cara et al. [26], which is sometimes also referred to as a ROH-based relationship matrix. In our study, $\mathbf{R}$ indicates whether animals are inbred at the same genomic positions, i.e. have ROH at the same genomic positions, whereas the segment-based relationship matrix indicates the probability that, if you mated two animals, the offspring would carry ROH. A more detailed explanation of how $\mathbf{R}$ was calculated, including a numerical example, is provided in [Additional file 2: Tables S1 and S2].

Goodness-of-fit of Models A, AD and ADR were compared using maximum likelihood (ML) ratio tests. Test statistics were defined as two times the difference between the maximum log likelihood of a reduced model (e.g. Model A) and that of a full model (e.g. Model AD). Approximate P-values were calculated as $0.5(1-\mathrm{Pr}\left({\chi }_{1}^{2}\le T\right))$, where $T$ was the test statistic.

Variance components were obtained from the mtg2 output. Relative variance components and corresponding standard errors were calculated using the delta method in mtg2 [23]. For example, the relative dominance variance was calculated as ${\sigma }_{D}^{2}/{\sigma }_{P}^{2}$, where ${\sigma }_{P}^{2}$ is the phenotypic variance (which excluded ${\sigma }_{HYS}^{2}$).

To estimate additive effects ($\widehat{{\varvec{\upalpha}}}$), dominance effects ($\widehat{\mathbf{d}}$) and ROH effects ($\widehat{\mathbf{r}}$) per SNP, the polygenic additive effects ($\widehat{\mathbf{u}}$), dominance deviations ($\widehat{\mathbf{v}}$) and ROH deviations ($\widehat{\mathbf{w}}$) were back-solved using the *compute SNP-effects* program of *calc_grm* [24], according to:

$$\widehat{{\varvec{\upalpha}}}=\frac{{\mathbf{Z}}^{\mathbf{^{\prime}}}{\mathbf{G}}^{-1}\widehat{\mathbf{u}}}{{\sum }_{i}2{p}_{i}{q}_{i}},$$

$$\widehat{\mathbf{d}}=\frac{{\mathbf{H}}^{\mathbf{^{\prime}}}{\mathbf{D}}^{-1}\widehat{\mathbf{v}}}{{\sum }_{i}{\left(2{p}_{i}{q}_{i}\right)}^{2}},$$

$$\widehat{\mathbf{r}}=\frac{{\mathbf{M}}^{\mathbf{^{\prime}}}{\mathbf{R}}^{-1}\widehat{\mathbf{w}}}{{\sum }_{i}{p}_{i}^{*}{q}_{i}^{*}},$$

where all parameters are defined as before. Note that for additive and dominance effects, ${p}_{i}$ and ${q}_{i}$ were allelic frequencies, whereas for ROH effects ${p}_{i}^{*}$ and ${q}_{i}^{*}$ were the frequencies of a SNP being in a ROH or not. The back-solving procedure was verified, by recalculating polygenic effects from the back-solved SNP effects.

Note that the dominance deviations ($\widehat{\mathbf{v}}$) and dominance SNP effects ($\widehat{\mathbf{d}}$) did not include directional dominance, because the mean dominance was already absorbed by the fixed regression on genome-wide homozygosity. The mean dominance effect across loci can be calculated as $-b/{N}_{SNP}$, where $b$ is the regression coefficient for genome-wide homozygosity and ${N}_{SNP}$ is the number of SNPs [27, 28]. In this study, we report the ${\sigma }_{D}^{2}$ and the dominance effects as obtained from GREML and back-solving output (thus, excluding mean dominance). However, we also computed the mean dominance effect (i.e. $-b/{N}_{SNP}$) and investigated the effect of correcting ${\sigma }_{D}^{2}$ for this mean dominance effect (see Discussion).

### Single SNP GWAS

A single SNP GWAS was performed to estimate additive, dominance and ROH effects as fixed effects per SNP. For this purpose, GREML Model A was extended by adding a fixed additive, dominance and ROH effect at a specific SNP. For each SNP, the following model was run with Snappy [29] in Wombat [30]:

$$\mathbf{y}=\mathbf{j}\alpha +\mathbf{k}d+\mathbf{l}r+\mathbf{X}\mathbf{b}+\mathbf{Q}\mathbf{c}+\mathbf{u}+\mathbf{e},$$

where $\mathbf{j}$ is a vector with allele counts (coded as 0, 1, and 2 for genotypes *BB*, *AB*, and *AA*); $\alpha$ is the additive effect; $\mathbf{k}$ is a vector with heterozygosity status (coded as 0, 1, and 0 for genotypes *BB*, *AB*, and *AA*); $d$ is the dominance effect; $\mathbf{l}$ is a vector with ROH status (coded as 1 when the SNP was in a ROH, or 0 otherwise); and $r$ is the ROH-effect. The other parameters are defined as in GREML Model A.

Solutions and t-statistics were obtained from the output, and corresponding P-values were computed. Genomic inflation was assessed using QQplots and genomic inflation factors. The latter were computed as the ratio of the observed median χ^2^ statistic over the expected median of the χ^2^ distribution under the null hypothesis [31]. To account for multiple testing, P-values were adjusted with the *p.adjust()* function in R by applying the approach of Benjamini and Hochberg [32]. A genome-wide false discovery rate (FDR) of 10% was used as a threshold to declare associations as being significant.

## Results

### Homozygosity and ROH-coverage along the genome

**Fig. 1:**
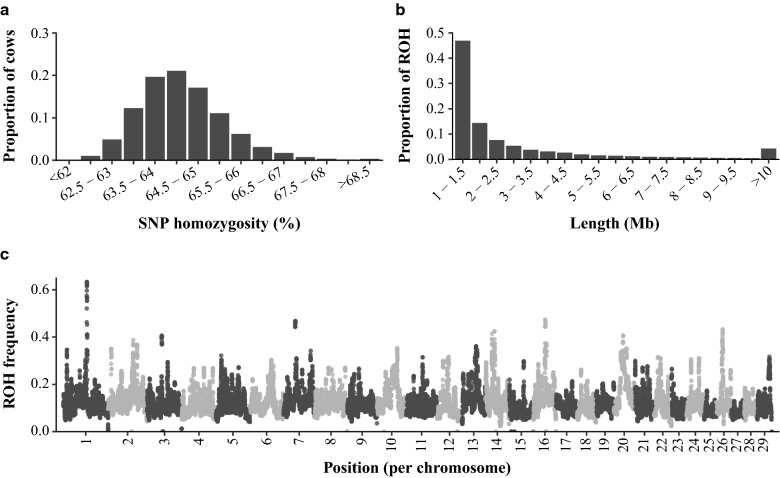
Summary statistics of SNP homozygosity and regions of homozygosity (ROH) across all cows: (**a**) Distribution of genome-wide SNP homozygosity; (**b**) Distribution of ROH length; (**c**) Frequency of each SNP being in a ROH by genomic position

Genome-wide SNP homozygosity of cows approximately followed a normal distribution with a mean of 64.4% and a standard deviation of 1.0% (Fig. 1a). In total, 3,910,969 ROH were identified. As expected, these ROH followed approximately an exponential distribution in terms of length (Fig. 1b), with short ROH being more abundant than long ROH. The frequency of a SNP being in a ROH was on average 11.5% [for distribution, see Additional file 1: Figure S2] and differed along the genome (Fig. 1c). Chromosomes 10, 16 and 20 had the highest ROH-frequency. The highest local peak was observed on chromosome 1, with a ROH-frequency of up to 63.3%. Sixty-two SNPs were never in a ROH. These SNPs were mostly located at the start or end of chromosomes.

The homozygosity status and ROH status partly overlapped. Of all SNPs across all individuals, 11.3% were both homozygous and in a ROH, 53.1% were homozygous but not in a ROH, 0.2% were heterozygous and in a ROH, and the remaining 35.4% were heterozygous and not in a ROH.

### Genome-wide inbreeding depression from GREML models

Genome-wide homozygosity had an unfavourable effect on all evaluated traits and across all GREML models (Table 1). For example, a 1% increase in homozygosity in Model A was associated with a decrease in 305-d milk yield of 99.6 kg (SE = 5.2), an increase in calving interval of 1.1 days (SE = 0.4) and an increase in SCS400 of 2.3 units (SE = 0.7). These unfavourable effects of genome-wide homozygosity reflect the presence of (favourable) directional dominance. For example, the mean dominance effect of a SNP in Model A was 0.13 kg for milk yield, − 0.0015 days for calving interval and − 0.0030 units in SCS400 (Table 1). Estimated effects of genome-wide inbreeding depression were similar across Models A, AD and ADR.

**Table 1:** Effect of a 1% increase in genome-wide homozygosity ($b$), and mean dominance effect per SNP ($-b/{N}_{SNP}$), for three models and nine traits

Trait	${N}_{cows}$	Model A		Model AD		Model ADR	
		$b$(SE)	$-b/{N}_{SNP}$	$b$(SE)	$-b/{N}_{SNP}$	$b$(SE)	$-b/{N}_{SNP}$
MY	38,778	− 99.6 (5.2)	0.1322	− 98.7 (6.1)	0.1310	− 97.8 (6.7)	0.1298
FY	38,778	− 4.10 (0.20)	0.0054	− 4.04 (0.23)	0.0054	− 4.01 (0.27)	0.0053
PY	38,778	− 3.49 (0.17)	0.0046	− 3.45 (0.20)	0.0046	− 3.42 (0.23)	0.0045
CI	34,864	1.11 (0.35)	− 0.0015	1.11 (0.35)	− 0.0015	1.11 (0.38)	− 0.0015
ICF	34,937	0.20 (0.15)	− 0.0003	0.21 (0.16)	− 0.0003	0.21 (0.17)	− 0.0003
IFL	34,937	0.79 (0.30)	− 0.0011	0.79 (0.30)	− 0.0010	0.79 (0.30)	− 0.0010
CR (%)	34,774	− 0.68 (0.19)	9.0E–06	− 0.68 (0.19)	9.0E–06	− 0.68 (0.19)	9.0E–06
SCS150	38,301	1.09 (0.69)	− 0.0015	1.08 (0.70)	− 0.0015	1.09 (0.71)	− 0.0014
SCS400	37,068	2.28 (0.67)	− 0.0030	2.26 (0.70)	− 0.0030	2.26 (0.70)	− 0.0030

Model A, additive model; AD, additive + dominance model; ADR, additive + dominance + ROH model

MY, 305-day milk yield (kg); FY, 305-day fat yield (kg); PY, 305-day protein yield (kg); CI, calving interval (days); ICF, interval calving to first insemination (days); IFL, interval first to last insemination (days); CR: conception rate (%); SCS150 somatic cell score day 5 to 150 (1000 + 100* [log2 of cells/mL]); SCS400: somatic cell score day 151 to 400 (1000 + 100*[log2 of cells/mL])

### Variance components and goodness of fit of GREML models

Additive genetic variance was observed for all traits (Table 2). In Model A, heritability estimates ranged from 2.36% (SE = 0.32%) for conception rate to 41.16% (SE = 0.81%) for milk yield. Heritability estimates were approximately identical across Models A, AD and ADR.

**Table 2:** Estimated variance components for three GREML models and nine traits, with standard errors in parentheses

Model	Parameter	Trait								
		MY	FY	PY	CI	ICF	IFL	CR	SCS150	SCS400
A	${\sigma }_{P}^{2}$	1356452	1813.04	1269.15	4215.19	717.658	3056.16	12.6991	17693.4	16336.5
	${\sigma }_{A}^{2}/{\sigma }_{P}^{2}$(%)	41.16 (0.81)	33.34 (0.82)	30.98 (0.81)	5.02 (0.43)	6.41 (0.49)	3.13 (0.35)	2.36 (0.32)	9.26 (0.54)	12.0 (0.60)
AD	${\sigma }_{P}^{2}$	1356134	1812.49	1268.93	4215.18	717.690	3056.15	12.6991	17693.82	16336.80
	${\sigma }_{A}^{2}/{\sigma }_{P}^{2}$(%)	41.13 (0.81)	33.31 (0.82)	30.95 (0.81)	5.02 (0.43)	6.41 (0.49)	3.13 (0.35)	2.35 (0.32)	9.26 (0.54)	11.94 (0.60)
	${\sigma }_{D}^{2}/{\sigma }_{P}^{2}$(%)	0.77 (0.28)	0.90 (0.31)	0.87 (0.32)	0.00 (0.38)	0.35 (0.41)	0.09 (0.39)	0.04 (0.40)	0.16 (0.35)	0.34 (0.36)
	${\sigma }_{A}^{2}/{\sigma }_{G}^{2}$(%)	98.17 (0.66)	97.36 (0.89)	97.27 (0.99)	99.93 (7.63)	94.76 (5.78)	97.23 (11.89)	98.52 (16.45)	98.30 (3.66)	97.22 (2.87)
	${\sigma }_{D}^{2}/{\sigma }_{G}^{2}$(%)	1.83 (0.66)	2.64 (0.89)	2.73 (0.99)	0.07 (7.63)	5.24 (5.78)	2.77 (11.89)	1.48 (16.45)	1.70 (3.66)	2.78 (2.87)
ADR	${\sigma }_{P}^{2}$	1355963	1812.48	1268.69	4215.08	717.624	3056.15	12.6991	17693.8	16336.8
	${\sigma }_{A}^{2}/{\sigma }_{P}^{2}$(%)	41.10 (0.81)	33.27 (0.82)	30.90 (0.81)	5.01 (0.43)	6.38 (0.49)	3.13 (0.35)	2.35 (0.32)	9.25 (0.54)	11.94 (0.60)
	${\sigma }_{D}^{2}/{\sigma }_{P}^{2}$(%)	0.51 (0.32)	0.54 (0.32)	0.52 (0.36)	0*	0.14 (0.47)	0.09 (0.39)	0.04 (0.40)	0.14 (0.40)	0.34 (0.36)
	${\sigma }_{ROH}^{2}/{\sigma }_{P}^{2}$(%)	0.17 (0.11)	0.25 (0.12)	0.24 (0.13)	0.07 (0.12)	0.13 (0.14)	0*	0*	0.01 (0.12)	0*
	${\sigma }_{A}^{2}/{\sigma }_{G}^{2}$(%)	98.38 (0.68)	97.68 (0.92)	97.62 (1.02)	98.53 (2.30)	95.94 (6.11)	97.23 (11.93)	98.52 (16.76)	98.39 (3.74)	97.22 (2.87)
	${\sigma }_{D}^{2}/{\sigma }_{G}^{2}$(%)	1.22 (0.75)	1.60 (1.01)	1.63 (1.12)	0*	2.09 (6.91)	2.77 (11.89)	1.48 (16.45)	1.46 (4.20)	2.78 (2.87)
	${\sigma }_{ROH}^{2}/{\sigma }_{G}^{2}$(%)	0.40 (0.26)	0.72 (0.37)	0.75 (0.40)	1.47 (2.25)	1.97 (2.21)	0*	0*	0.15 (1.29)	0*

Model A, additive model; AD, additive + dominance model; ADR, additive + dominance + ROH model

${\sigma }_{P}^{2}$, phenotypic variance (excluding the herd-year-season variance); ${\sigma }_{A}^{2}$, additive genetic variance; ${\sigma }_{D}^{2}$, dominance variance; ${\sigma }_{ROH}^{2}$, ROH variance; ${\sigma }_{G}^{2}$, genetic variance (${\sigma }_{A}^{2}+{\sigma }_{D}^{2}$ for model AD, and ${\sigma }_{A}^{2}+{\sigma }_{D}^{2}+{\sigma }_{ROH}^{2}$ for model ADR)

MY, 305-day milk yield (kg); FY, 305-day fat yield (kg); PY, 305-day protein yield (kg); CI, calving interval (days); ICF, interval calving to first insemination (days); IFL, interval first to last insemination (days); CR, conception rate (%); SCS150 somatic cell score day 5 to 150 (1000 + 100*[log2 of cells/mL]); SCS400: somatic cell score day 151 to 400 (1000 + 100*[log2 of cells/mL])

^*^The corresponding variance component was fixed to 0 (because its initial estimate was slightly negative)

In Model AD, 0.8 to 0.9% of the phenotypic variance for yield traits and less than 0.4% of the phenotypic variance for all other traits was attributable to dominance. When expressed as part of the total genetic variance, dominance variance explained on average 2.36% of the genetic variance in Models AD (with a range of 0.07 to 5.24% across traits). The small contribution of dominance was also reflected by the goodness-of-fit of the different models. When moving from Model A to AD, the maximum log likelihood increased significantly (*P* < 0.05) only for yield traits (Table 3). For these yield traits, the maximum log likelihood further increased (*P* < 0.05) when moving to Model ADR. In Model ADR, the relative ROH variance for yield traits was approximately 0.2% (Table 2), while the relative dominance variance was lower than that in Model AD (i.e. 0.5% instead of 0.8%).

**Table 3:** Comparison of goodness–of-fit of different GREML models for nine traits

Trait	Log-likelihood of Model A	Difference in log-likelihood		P-value	
		AD-A	ADR-AD	AD vs A	ADR vs AD
MY	− 287087.83	4.172	1.437	0.002	0.045
FY	− 161337.92	5.014	2.355	< 0.001	0.015
PY	− 155089.55	4.141	2.120	0.002	0.020
CI	− 162356.33	0.000	0.236	0.500	0.246
ICF	− 132424.64	0.382	0.436	0.191	0.175
IFL	− 157131.13	0.026	0.000	0.410	0.500
CR	− 141255.38	0.004	0.000	0.465	0.500
SCS150	− 205365.12	0.107	0.007	0.322	0.453
SCS400	− 196930.13	0.464	0.000	0.168	0.500

Model A, additive model; AD, additive + dominance model; ADR, additive + dominance + ROH model

MY, 305-day milk yield; FY, 305-day fat yield; PY: 305-day protein yield; CI, calving interval; ICF, interval calving to first insemination; IFL, interval first to last insemination; CR, conception rate; SCS150 somatic cell score day 5 to 150; SCS400, somatic cell score day 151 to 400

The herd-year-season variance (data not shown) was similar across Models A, AD and ADR and was highest for yield traits (6.7% to 9.8% of total variance) and for the interval between calving and first insemination (5.8% of total variance). The latter trait is known to be strongly influenced by farmers’ decision.

### Comparison of GREML and GWAS effects

**Fig. 2:**
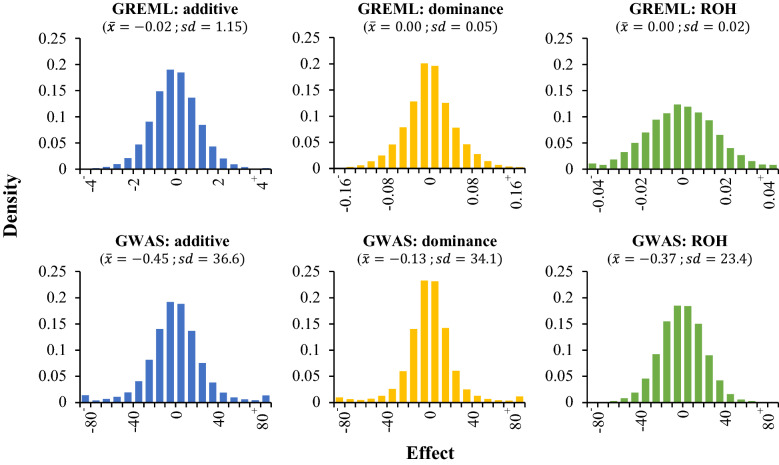
Distributions of SNP effects for 305-day milk yield (kg), estimated by GREML and single SNP GWAS. The mean ($\bar{x}$) and standard deviation ($sd$) of the effects are shown. Note that distributions were truncated such that the first and last bar represent “smaller than” and “bigger than” classes (i.e. the range was larger than shown here). Also note that the dominance effects shown here do not include the mean dominance effect that was absorbed by the fixed regression on genome-wide homozygosity

Estimated additive, dominance and ROH effects from back-solving in GREML and from single SNP GWAS models were approximately normally distributed with a mean of zero (Fig. 2). The range and standard deviation of GWAS effects were substantially larger than those of GREML effects. For example, additive effects for milk yield estimated by GWAS ranged from − 1069 to 1020 kg with a standard deviation of 36.6 kg, whereas those estimated by GREML ranged from − 25.7 to 17.8 kg with a standard deviation of 1.1 kg. The difference between GWAS and GREML estimates was larger for dominance and ROH effects than for additive effects. For example, dominance effects for milk yield estimated by GWAS ranged from − 1038 to 1120 kg with a standard deviation of 34.1 kg, whereas those estimated by GREML ranged from − 0.25 to 0.25 kg with a standard deviation of 0.05 kg.

**Fig. 3:**
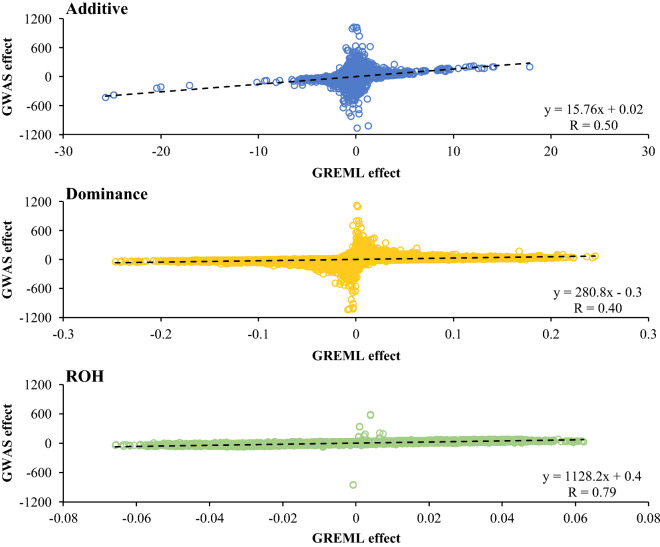
Scatterplots comparing SNP effects for 305-day milk yield (kg) estimated by GREML and single SNP GWAS. The dashed line is a linear trendline. The regression equation corresponding to this line and the Pearson correlation coefficient (R) are shown. Note that the dominance effects shown here do not include the mean dominance effect that was absorbed by the fixed regression on genome-wide homozygosity

There was a moderate correlation between SNP effects estimated by GREML and GWAS. For milk yield, for example, this correlation was 0.50 for additive effects, 0.40 for dominance effects and 0.79 for ROH effects. For additive and dominance effects, many SNPs had large (absolute) GWAS effects but a GREML effect close to zero (Fig. 3).

### Additive effects across the genome

**Fig. 4:**
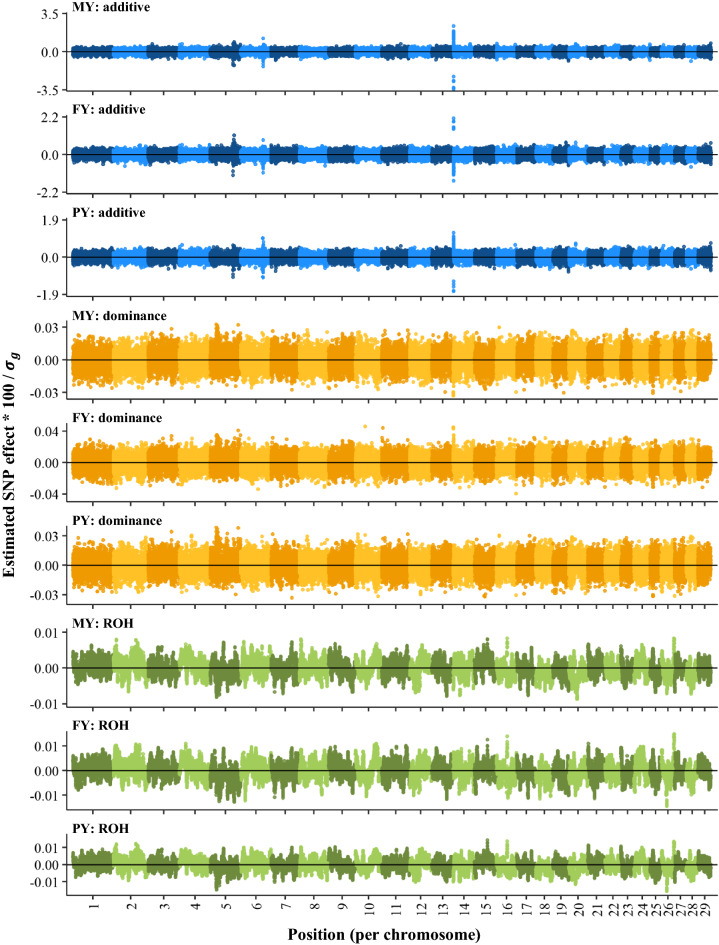
Additive, dominance and ROH effects for yield traits, estimated by GREML (model ADR) with back-solving. MY: 305-day milk yield (kg); FY: 305-day fat yield (kg); PY: 305-day protein yield (kg). Effects were multiplied by 100 and divided by the genetic standard deviation (${\sigma }_{g}$) of the corresponding trait. Note that the dominance effects shown here do not include the mean dominance effect that was absorbed by the fixed regression on genome-wide homozygosity

**Fig. 5:**
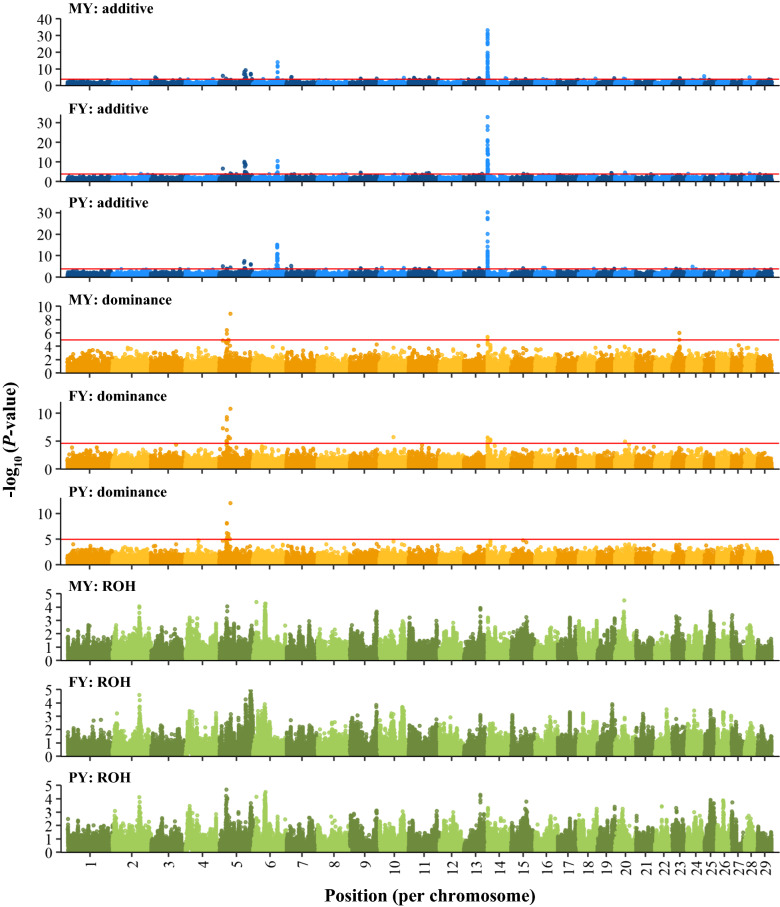
Statistical significance of additive, dominance and ROH effects for yield traits from single SNP GWAS. MY: 305-day milk yield; FY: 305-day fat yield; PY: 305-day protein yield. The horizontal red line is a threshold based on 10% false-discovery rate (absence of this line implies that all effects were below the threshold). The y-axis for MY additive effects was truncated at 40; in the peak on chromosome 14, there were 6 SNPs with a -log_10_(P-value) ranging from 40 to 94

Estimated additive genetic effects followed a similar pattern as that reported for other GWAS. Manhattan plots of SNP effects for yield traits, obtained by back-solving from the GREML ADR Models, are in Fig. 4. As expected, SNPs with the largest additive effects for yield traits were located between 0 and 1 Mb on chromosome 14, surrounding the *DGAT1* gene [33]. The effects in this region were antagonistic, i.e. alleles that were favorable for milk and protein yields were unfavorable for fat yield. Two other regions had major additive effects on all yield traits, one on chromosome 5 (approximately 88.2 to 88.5 Mb), surrounding the *ABCC9* gene [33], and one on chromosome 6 (near 87 Mb), surrounding the *GC* gene [33]. For protein yield, there was an additional peak on chromosome 6 (approximately 85.4 to 85.7 Mb), which included the casein cluster, i.e. *CSN1*, *CSN2* and *CSN3* [33]. The abovementioned additive peaks also passed the 10% FDR threshold in the GWAS (Fig. 5). Genomic inflation factors of the GWAS for additive and dominance effects were all lower than 1.1, suggesting that there was no major inflation of P-values for these effects [see Additional file 3: Figure S3].

The peaks of GREML additive effects were less pronounced for fertility and udder health traits than for yield traits [see Additional file 4: Figure S4]. In addition, for fertility and udder traits, fewer SNPs showed a significant additive association in the GWAS [see Additional file 5: Figure S5]. The most notable region with significant associations was a region on chromosome 19 for SCS400. This region consisted of two narrow subpeaks (one around 54.6 Mb and one around 55.3 Mb). For the interval between calving and first insemination (ICF), various significant additive associations were detected in the GWAS. The most notable region, which was also identified by the GREML approach, was on chromosome 28 (near 35.8 Mb).

### Dominance and ROH effects along the genome

GREML-based dominance effects showed less pronounced peaks than additive effects (Fig. 4) [see also Additional file 4: Figure S4]. In the GWAS, there were also fewer significant dominance associations than significant additive associations (Fig. 5) [see also Additional file 5: Figure S5].

For yield traits, the most notable region with large favorable dominance effects in the GREML and with significant dominance associations in the GWAS was located on chromosome 5 (Figs. 4 and 5). This region was rather wide, with significant associations between 13 and 40 Mb and the largest effects between 24 and 28 Mb [see Additional file 6: Figures S6 and S7]. In addition to the region on chromosome 5, two other peaks passed the 10% FDR in the GWAS, one near *DGAT1* for milk and fat yields and one on chromosome 23 (near 25.2 Mb) for milk yield.

For fertility and udder health traits, there were very few significant dominance associations in the GWAS (Additional file 5). The only significant SNPs were found for the interval between calving and first insemination (ICF) and these SNPs did not cluster in peaks.

ROH effects showed many narrow peaks for all traits, both with GREML and GWAS (Figs. 4 and 5) [see also Additional file 4: Figure S4 and Additional file 5: Figure S5]. However, none of the ROH effects in the GWAS passed the 10% FDR.

## Discussion

The objective of this study was to obtain a better understanding of (differences in) inbreeding depression across the genome of Dutch Holstein Friesian dairy cattle. To fulfil this objective, first we estimated genome-wide inbreeding depression and estimated additive, dominance and ROH variance components with GREML models. Then, we investigated dominance and ROH effects along the genome for yield, fertility and udder health traits, using GREML (with back-solving) and a single SNP GWAS.

### Genome-wide inbreeding depression

Genome-wide SNP homozygosity had an unfavourable effect on all traits, indicating the presence of directional dominance (Table 1). The estimated effects were comparable in size to those previously reported for similar traits in Holstein Friesian dairy cattle [2, 3, 8]. When comparing inbreeding depression estimates across studies, it is important to consider the variance of underlying inbreeding measures [34]. For example in this study, the effect of a 1% increase in SNP homozygosity on milk yield (of approximately − 99 kg) may seem larger than the effect of a 1% increase in ROH-based inbreeding that we previously reported (of approximately − 36 kg [8]), but this difference can be largely explained by the different scale of the inbreeding measures. In this study, SNP homozygosity had a mean of 64.4% and a standard deviation of 1.0%, whereas ROH-based inbreeding in our previous study had a mean of 12.3% and a standard deviation of 2.7% [8]. Therefore, a 1% increase in SNP homozygosity captures a larger effect at the population level than a 1% increase in ROH-based inbreeding. To illustrate this effect of scale, previously we compared the phenotypes of highly inbred cows and lowly inbred cows and showed that different inbreeding measures may result in similar inbreeding depression at the population level, in spite of the difference in estimated regression coefficients [8].

### Dominance and ROH variance

Estimates of dominance and ROH variances were small and differed significantly from zero for yield traits only (Tables 2 and 3). Dominance and ROH variances explained less than 1% of the phenotypic variance, and less than 5% of the genetic variance. When we ran models excluding dominance, i.e. Models AR, the ROH variance also explained less than 1% of the phenotypic variance [see Additional file 7: Table S3]. The maximum log-likelihoods of Models AR were significantly higher than those of Models A for yield traits only [see Additional file 7: Table S4].

Many other studies have estimated dominance variance in Holstein Friesian dairy cattle. Here, we use “relative dominance variance” for the ratio of dominance variance over phenotypic variance. Note that in the literature the term “dominance heritability” is also sometimes used for the same ratio [14, 35, 36]. Estimates of relative dominance variance based on pedigree relationship matrices in Holstein cattle typically range from 1 to 5% [37–40], although a few studies suggest a larger contribution of dominance effects [41, 42]. Estimates based on genomic relationship matrices are similar to, or slightly higher, than pedigree-based estimates (Table 4).

**Table 4:** Estimates of relative dominance variance from various studies that used genomic relationship matrices

Study	Density	Accounted for GW ID^a^	Relative dominance variance
Aliloo et al. [14]	632 k (imputed)	Yes	≤ 1% for yield traits
			1% for calving interval
Aliloo et al. [14]	632 k (imputed)	No	3 to 4% for yield traits
			1% for calving interval
Sun et al. [35]	50 k (imputed)	Yes, in precorrection of phenotypes	3 to 4% for yield traits
			1% for SCS
			0% for daughter pregnancy rate
Jiang et al. [43]^b^	50 k (imputed)	No	7 to 13% for yield traits
			0 to 15% for fertility traits
			9% for SCS
Alves et al. [44]	41 k (imputed)	No	0 to 4% for fertility traits
Mao et al. [45]	36 k	No	7% for interval first-last insemination
			4% for number of inseminations

^a^GW ID: genome-wide inbreeding depression

^b^In this particular study, an imprinting effect was also fitted. All other studies used AD models

Our relative dominance variance estimates tend to be a bit lower than most estimates from the literature. One explanation is that we corrected for genome-wide inbreeding depression in our GREML models (as discussed in the next section). Another reason for low dominance variance may be the limited SNP density that we used, although most other studies used similar densities (Table 4). It is well known that the additive variance captured by a SNP depends on ${r}^{2}$ (with $r$ being the correlation between the SNP and a QTL), while the dominance variance captured by the SNP depends on ${r}^{4}$ [46]. In other words, the detection of dominance effects relies more on high LD than detection of additive effects. Thus, the detection of dominance effects would benefit substantially from a higher SNP density. In addition to SNP density and the inclusion of a regression on genome-wide homozygosity, many other factors may explain differences in relative dominance variance across studies. These factors include differences in trait definition, differences in the way phenotypes are (pre)corrected for fixed effects, differences in how the dominance relationship matrix is calculated and population-specific differences [13, 47].

In this study, we introduced a ROH-based relationship matrix to estimate a ROH-based variance component. For yield traits, Model ADR showed a better fit than Model AD (Table 3), which suggests some benefit of including ROH effects. This benefit appeared to be due to an overall redistribution of variance components and could not be easily explained. In fact, the proportion of variance explained by dominance in Model AD was slightly higher than the combined proportion of variance explained by dominance and ROH effects in Model ADR (Table 2). The dominance variance from the AD model appeared to be split over dominance and ROH components in Model ADR, suggesting that dominance and ROH effects partly captured the same variation (which might be due to collinearity between the $\mathbf{D}$ and $\mathbf{R}$ matrices, see discussion on Orthogonality of $\mathbf{G}$, $\mathbf{D}$ and $\mathbf{R}$). However, differences in variance components across models were small and may have also been due to random sampling.

### Accounting for directional dominance when estimating dominance variance with GREML

In our GREML models, we corrected for genome-wide inbreeding depression by including a fixed regression on genome-wide SNP homozygosity. This correction is important to ensure that the model assumptions of a mean dominance effect of zero ($\mathrm{E}\left[\mathrm{v}\right]=0$) and of no covariance between additive and dominance effects ($\mathrm{cov}[\mathrm{u},\mathrm{v}]=0$) hold, and to prevent the dominance variance from being inflated [10, 28, 47]. Indeed, when we removed the fixed regression on genome-wide homozygosity from Model AD, the relative dominance variance for yield traits increased to approximately 3% (as compared to 0.8%), which are values similar to those reported by Aliloo et al. [47]. In addition, the mean back-solved dominance effect was no longer zero, but slightly favourable. The mean back-solved dominance effect for milk yield, for example, was 0.05 kg when not accounting for genome-wide homozygosity (as compared to 0.0005 kg when accounting for genome-wide homozygosity). Note that this 0.05 kg is smaller than the 0.13 kg from the fixed regression on genome-wide homozygosity (Table 1), which may be explained by shrinkage on the mean dominance when it was part of the random effect.

When a fixed regression on genome-wide SNP homozygosity is included in an AD model, the mean dominance effect across all loci is absorbed by this regression [27, 28]. Consequently, the ${\sigma }_{D}^{2}$ of such models is expected to be underestimated. Namely, the ${\sigma }_{D}^{2}$ of such models captures only the deviations of dominance effects (${d}_{i}$) at individual loci from the mean dominance effect ($\bar{d}$) across all loci, ${\sigma }_{D}^{2}={\sum }_{i}{(2{p}_{i}{q}_{i}({d}_{i}-\bar{d}))^{2}},$ while the full dominance variance equals ${\sigma }_{{D}_{FULL}}^{2}={\sum }_{i}{\left(2{p}_{i}{q}_{i}{d}_{i}\right)^{2}}$. Thus, to obtain ${\sigma }_{{D}_{FULL}}^{2}$, a component related to the mean dominance effect across all loci should be added to the ${\sigma }_{D}^{2}$ from the GREML output. This additional component can be derived as:

$${\sigma }_{{D}_{FULL}}^{2}={\sum }_{i}{\left(2{p}_{i}{q}_{i}{d}_{i}\right)^{2}}$$

$$={\sum }_{i}{\left(2{p}_{i}{q}_{i}({d}_{i}-\bar{d})+2{p}_{i}{q}_{i}\bar{d} \right)^{2}}$$

$$={\sum }_{i}{(2{p}_{i}{q}_{i}({d}_{i}-\bar{d}))^{2}}+{\sum }_{i}{(2{p}_{i}{q}_{i}\bar{d})^{2}}$$

$$= {\sigma }_{D}^{2}+N\overline{{(2pq)}^{2}}{\bar{d}}^{2},$$

where ${\sigma }_{{D}_{FULL}}^{2}$ is the full dominance variance, ${\sigma }_{D}^{2}$ is the dominance variance obtained from the GREML output, $N$ is the number of SNPs, $\overline{{(2pq)}^{2}}$ is the mean squared expected heterozygosity, and ${\bar{d}}^{2}$ is the squared mean dominance effect, where $\bar{d}$ can be obtained from the regression on genome-wide homozygosity. Note that, in the third line of the derivation above, a cross-product has disappeared because $\sum ({d}_{i}-\bar{d})=0.$

To quantify the difference between ${\sigma }_{D}^{2}$ and ${\sigma }_{{D}_{FULL}}^{2}$, we calculated ${\sigma }_{{D}_{FULL}}^{2}$ for Model AD, applying the reasoning above. The additional component, $N\overline{{(2pq)}^{2}}{\bar{d}}^{2}$, was relatively small compared to ${\sigma }_{D}^{2}$. For milk yield, for example, $N\overline{{(2pq)}^{2}}{\bar{d}}^{2}$ equalled 189 kg^2^, whereas ${\sigma }_{D}^{2}$ equalled 10,377 kg^2^. Consequently, the relative dominance variance increased only marginally when accounting for the additional $N\overline{{(2pq)}^{2}}{\bar{d}}^{2}$ component (e.g. from 0.77 to 0.78% for milk yield).

In Model ADR, it was assumed that ROH effects were distributed as ${\sim N(\mathbf{0},\mathbf{R}\sigma }_{ROH}^{2}$). This assumption may not hold, because of a potential average genome-wide ROH-effect being different from zero (similar to the genome-wide dominance effect). However, since genome-wide SNP homozygosity and genome-wide ROH coverage (the ${F}_{ROH}$) are highly correlated [8], we expected that the inclusion of genome-wide homozygosity would largely correct for a genome-wide ROH effect. In Model ADR, the means of the back-solved ROH effects were approximately zero (e.g. − 0.0007 for milk yield), suggesting that the fixed effect for genome-wide homozygosity indeed removed the mean ROH effect.

### Orthogonality of $\mathbf{G}$, $\mathbf{D}$ and $\mathbf{R}$

In the GREML models in this study, the $\mathbf{G}$ and $\mathbf{D}$ were parameterized according to Vitezica et al. [13], ensuring orthogonality between these matrices. $\mathbf{R}$ was constructed using VanRaden’s method 1 [25], with ROH-status of SNPs as input instead of genotype status. Since the ROH-status of a SNP partly depends on its genotype status (e.g. a SNP typically must be homozygous to be in a ROH), some collinearity was expected between $\mathbf{R}$ on the one hand, and $\mathbf{G}$ and $\mathbf{D}$ on the other hand. To check for collinearity, correlations between off-diagonals of the different relationship matrices were calculated. These correlations equalled 0.03 between $\mathbf{G}$ and $\mathbf{D}$, 0.18 between $\mathbf{G}$ and $\mathbf{R}$, and 0.44 between $\mathbf{D}$ and $\mathbf{R}$. Correlations between variance component estimates were also calculated, using the average information matrix from Models ADR, and equalled approximately 0 between ${\sigma }_{G}^{2}$ and ${\sigma }_{D}^{2}$, 0 between ${\sigma }_{G}^{2}$ and ${\sigma }_{ROH}^{2}$, and 0.41–0.48 between ${\sigma }_{D}^{2}$ and ${\sigma }_{ROH}^{2}$ [see Additional file 8: Table S5]. This suggests that there was moderate collinearity between $\mathbf{R}$ and $\mathbf{D}$ and that it was difficult to disentangle the dominance and ROH variance components.

Although the moderate collinearity between $\mathbf{D}$ and $\mathbf{R}$ may have complicated the partitioning of variance components, the ranking of estimated SNP effects did not seem to be affected. Correlations between back-solved dominance effects of Models AD and ADR were above 0.998 for all traits. Similarly, correlations between additive effects of Models A, AD and ADR were above 0.999 for all traits.

Our objective was to investigate dominance and ROH effects along the genome, and the ranking of these effects did not seem to be affected by collinearity. Therefore, we believe that the collinearity had no effect on the results and conclusions of this study and decided not to perform a precorrection for orthogonality in our study. If similar analyses were to be performed in the future, orthogonality of $\mathbf{G}$, $\mathbf{D}$ and $\mathbf{R}$ could be ensured by a pre-correction of the indicator matrix underlying $\mathbf{R}$ (i.e. the $\mathbf{M}$ in this study), making it independent from the indicator matrices underlying $\mathbf{G}$ (i.e. $\mathbf{Z}$) and $\mathbf{D}$ (i.e. $\mathbf{H}$). This could be achieved by linear regression. For each SNP $i$, the corresponding vector in $\mathbf{M}$ (say ${\mathrm{M}}_{\mathrm{i}}$) could be regressed on the corresponding vectors of $\mathbf{Z}$ (i.e. ${\mathrm{Z}}_{\mathrm{i}}$) and $\mathbf{H}$ (i.e. ${\mathrm{H}}_{\mathrm{i}}$). The residuals of this linear regression could then be used as the new indicator variable (say ${\mathrm{M}}_{\mathrm{i}}^{*}$) to build a corrected $\mathbf{R}$-matrix. By virtue of least squares, the ${\mathrm{M}}_{\mathrm{i}}^{*}$ would be orthogonal to ${\mathrm{Z}}_{\mathrm{i}}$ and ${\mathrm{H}}_{\mathrm{i}}$.

### SNP effects estimated by GREML and single SNP GWAS

In this study, we estimated SNP effects with two approaches: GREML with back-solving and a single SNP GWAS. It is important to note that these approaches are equivalent when they are used for association mapping and when the models are strictly identical. As shown mathematically by Bernald Rubio et al. [48], the test statistics of GREML and of single SNP GWAS are equivalent if models are identical. These test statistics can be calculated as the estimates of effects divided by their standard deviation [48]. In this study, we did not compare the test statistics of the GREML and GWAS models, because of two software-related reasons: (1) the $\mathbf{D}$ and $\mathbf{R}$ matrices were not fitted in the single SNP GWAS, introducing a difference between GREML and GWAS models, and (2) the standard deviations of GREML effects were not available.

**Fig. 6:**
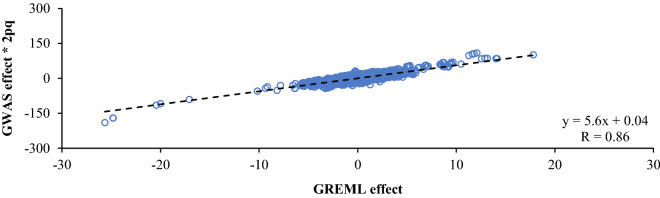
Scatterplot comparing additive effects for 305-day milk yield (kg) estimated by GREML and by GWAS with manual shrinkage. The GWAS effects were manually shrunk by multiplying them with $2pq$. The dashed line is a linear trendline. The regression equation corresponding to this line and the Pearson correlation coefficient (R) are shown

Although we did not compare test statistics, we did compare the effects of SNPs. Estimated effects of SNPs were much larger for the single SNP GWAS than for GREML and correlations between the effects of both approaches were moderate (Figs. 2 and 3). This can be explained by the fact that a single SNP GWAS estimates the effects for one SNP at a time as fixed effect without shrinkage, whereas GREML estimates the effects of all SNPs together as random effect with shrinkage. The magnitude of shrinkage depends on the standard error of the estimate of the effect, which in turn depends on the amount of data and the variance of the associated factor. There is more shrinkage for a factor that explains a smaller proportion of the variance [49]. This explains why the effects of GREML are so much smaller than those of the single SNP GWAS (Figs. 2 and 3), especially for dominance effects (which explained little variance) and ROH effects (which explained even less variance). Shrinkage can also explain why there were various SNPs with a large absolute additive and dominance effect for GWAS, but with a close to zero additive and dominance effect for GREML (Fig. 3). These SNPs all had a low minor allele frequency (MAF). For additive effects, the degree of shrinkage at a SNP (given a fixed sample size) depends on $2pq$ [49]. When we manually shrunk additive effects from the GWAS by multiplying them with $2pq$, the outliers indeed disappeared and the correlation between additive effects of GWAS and GREML increased from 0.50 to 0.86 (Fig. 6).

### Region-specific inbreeding depression

We found limited evidence for region-specific inbreeding depression based on 75 k SNP data for yield, fertility and udder health traits. For yield traits, we only found a few regions with large favourable dominance effects from GREML and significant dominance associations in the GWAS (Figs. 4 and 5). These regions were similar to those identified in previous studies. For example, in a recent large-scale GWAS with approximately 300 k US Holsteins and 60 k SNP data, Jiang et al. [11] also found the most significant dominance effects for yield traits on chromosome 5 between 24 and 28 Mb. The second most significant dominance peak for milk yield that they found was located on chromosome 23 (near 18 Mb). The latter peak was also observed in an earlier GWAS with 43 k Holsteins [43]. The peak that we observed on chromosome 23 (near 25 Mb) was not exactly at the same location, but close to the previously reported peak. We also found a significant dominance peak near the *DGAT1* gene on chromosome 14 for milk and fat yields. Significant dominance effects of *DGAT1* on milk and fat yields have been reported before [11, 50, 51]. For non-yield traits, we found very few significant dominance associations for fertility traits and no significant dominance associations for SCS, also similar to findings of Jiang et al. [11].

We detected no significant GWAS associations for ROH effects. Pryce et al. [2], in contrast, reported various candidate regions associated with inbreeding depression for yield traits and calving interval based on a single SNP GWAS for ROH status in US Holsteins. This may be explained by two differences in the approach used. First, we estimated the additive, dominance and ROH effects simultaneously, whereas Pryce et al. [2] had no dominance effect in the model. Second, Pryce et al. [2] used a threshold of -log10(P-value) of 3 to identify candidate regions and mentioned that the FDR was high. When we applied a threshold -log10(P-value) of 3 in the GWAS, we indeed found various significant ROH peaks for all traits (Fig. 5) and [see Additional file 5 Figure S5]. Some of these peaks were favourable, potentially indicating signatures of selection, whereas others were unfavourable, potentially indicating inbreeding depression. Also note that genomic inflation factors were approximately 1.3 for ROH effects [see Additional file 3 Figure S3], suggesting substantial inflation. Because the ROH effects did not pass the 10% FDR, we decided not to correct for this inflation.

In this and other studies in which a single SNP GWAS for ROH-status was performed [2, 16], the ROH-status of a SNP was set to 1 when the SNP was in a ROH, irrespective of which ROH it concerned. As a result, the estimated ROH-effect of a SNP was a pooled effect of many distinct ROH. This approach is in line with the reasoning behind inbreeding depression, namely that any homozygosity decreases performance (irrespective for which allele). However, it may be of interest to know which specific ROH is unfavourable. Fine-mapping of individual ROH effects is not straightforward due to the large number of distinct ROH (each having a low frequency). One possibility is to group ROH based on common core regions and then try to associate ROH-groups with the phenotype, as is sometimes done in humans (e.g. [52].). Alternatively, one could test each individual ROH, e.g. using the heuristic approach introduced by Howard et al. [53]. In the approach of Howard et al. [53], the mean phenotype of individuals with a specific ROH is compared to the mean phenotype of individuals without that ROH. A limitation of this approach is that it is computationally intensive, in spite of the various filtering criteria that can be used (such as minimum ROH-frequency). Consequently, the feasibility for datasets with many traits and large numbers of individuals is limited. Recently, this approach has been applied to smaller datasets (less than 10,000 individuals) in swine [53] and Canadian Holstein cattle [54], and the estimated effects reported by these studies are rather large. For example, the average effect of unfavourable ROH identified by Marras et al. [54] for 305-d milk yield in first parity cows, was -295 kg with a standard deviation of 105 kg. These effects are likely to be overestimated, because of statistical biases similar to those in a single SNP GWAS. In addition, there may be many false negatives due to the initial filtering steps and the use of significance thresholds to account for multiple testing. Despite these limitations, the identified unfavourable ROH-haplotypes and their effects could be used to build an inbreeding load matrix (ILM), which provides some information on the expected inbreeding load of the offspring of a particular mating [53]. This could be valuable in mating programs but is of less importance for selection schemes.

An important observation in this study was that dominance (and ROH) effects shrunk substantially when the fixed regression on homozygosity was included in GREML models. This was also observed by Aliloo et al. [47]. As a result, ROH and dominance variances were small (< 1% of phenotypic variance) and only significant for yield traits. This suggests that, after correcting for genome-wide inbreeding depression, fitting dominance and ROH effects (based on 75 k data) had little additional value. Overall, our findings suggest that, based on 75 k SNP data, the deleterious effect of homozygosity is quite evenly distributed across the genome and is well captured by genome-wide homozygosity in Holstein–Friesian dairy cattle. Based on these findings, there is little benefit of accounting for region-specific inbreeding depression in selection schemes.

## Conclusions

Inbreeding depression was observed for yield, fertility and udder health traits in Dutch Holstein Friesian cattle. However, after correcting for genome-wide homozygosity dominance and ROH effects explained very little variance in GREML models. A few regions with relatively large favourable dominance effects and significant dominance associations (based on 10% FDR) were identified for yield traits, based on both GREML and single SNP GWAS. Overall, however, inbreeding depression appeared to be distributed quite equally along the genome and was well captured by genome-wide homozygosity.

## Supplementary information

**Additional file 1: Figure S1.** Allele frequency distribution of SNPs, shown as number of SNPs per minor allele frequency (MAF) class of 1% (e.g. from 0 to 1%). **Figure S2.** ROH frequency distribution of SNPs, shown as number of SNPs per ROH frequency class of 1% (e.g. from 0 to 1%). The last class includes all SNPs with a ROH frequency above 35%.

**Additional file 2: Table S1.** Construction of the **R** matrix for the ADR-model: SNP coding for ROH status and ROH-frequency per SNP and example of genotypes and ROH-status for 30 SNPs and two animals (*j* and *k*) in Table S1. **Table S2.** Construction of the **R** matrix for the ADR-model: example of calculating the ROH-based relationship between two animals (*j* and *k*) based on their ROH-status (${x}_{ij}$ and ${x}_{ik}$) for the 30 SNPs in Table S1. Applying Eq. (S1), the ROH-based relationship between *j* and *k* is 3.0471/3.4829 = 0.8749. Note that the frequency of each SNP being in a ROH (${p}_{i}^{*}$) was assumed to be known.

**Additional file 3: Figure S3.** QQ-plots and genomic inflation factors for P-values corresponding to additive, dominance and ROH effects estimated by a single SNP GWAS for nine traits. MY: 305-day milk yield; FY: 305-day fat yield; PY: 305-day protein yield; CI: calving interval; ICF: interval calving to first insemination; IFL: interval first to last insemination; CR: conception rate; SCS150 somatic cell score day 5 to 150; SCS400: somatic cell score day 151 to 400.

**Additional file 4: Figure S4.** Additive, dominance and ROH effects for fertility and udder health traits, estimated by GREML model ADR with back-solving. CI: calving interval (d); ICF: interval calving to first insemination (d); IFL: interval first to last insemination (d); CR: conception rate (%); SCS150 somatic cell score day 5 to 150 (units); SCS400: somatic cell score day 151 to 400 (units). Effects were multiplied by 100 and divided by the genetic standard deviation (${\sigma }_{g}$) of the corresponding trait. Note that dominance effects for CI and ROH effects for IFL, CR, and SCS400 are not shown, because the corresponding variances were fixed to zero in the GREML (Table 2 of main text).

**Additional file 5: Figure S5.** Statistical significance of additive, dominance and ROH effects for fertility and udder health traits based on single SNP GWAS (*continued on next page*). The horizontal red line is a threshold based on 10% false-discovery rate (absence of this line indicates that all effects were below the threshold). CI: calving interval (d); ICF: interval calving to first insemination (d); IFL: interval first to last insemination (d); CR: conception rate (%); SCS150 somatic cell score day 5 to 150 (units); SCS400: somatic cell score day 151 to 400 (units).

**Additional file 6: Figure S6.** Dominance effects for yield traits, estimated by GREML (model ADR) with back-solving, for a region on chromosome 5 from 10 to 45 Mb. MY: 305-day milk yield (kg); FY: 305-day fat yield (kg); PY: 305-day protein yield (kg). Effects were multiplied by 100 and divided by the genetic standard deviation (${\sigma }_{g}$) of the corresponding trait. **Figure S7.** Statistical significance of dominance effects for yield traits, estimated by single SNP GWAS, for a region on chromosome 5 from 10 to 45 Mb. MY: 305-day milk yield.

**Additional file 7: Table S3.** Estimated variance components^1^ for additive + ROH model (AR) and nine traits^2^, with standard errors in parentheses. **Table S4.** Comparison of goodness of fit of different GREML models for nine traits.

**Additional file 8: Table S5.** Correlations between variance components estimates^1^ from the ADR model for nine traits. Correlations were calculated from the average information matrix from mtg2 output.

## Data Availability

All information supporting the results is included in the text, figures, tables and Additional files. The dataset is not publicly available due to commercial restrictions.
