# Supplementary material for: Inbreeding depression across the genome of Dutch Holstein Friesian dairy cattle
Source: Genet Sel Evol. 2020 Oct 28;52:64. doi: 10.1186/s12711-020-00583-1 (PMC7594306; doi:10.1186/s12711-020-00583-1)
Supplement: Supplementary file 1 — Additional file 1: Figure S1. Allele frequency distribution of SNPs, shown as number of SNPs per minor allele frequency (MAF) class of 1% (e.g. from 0 to 1%). Figure S2. ROH frequency distribution of SNPs, shown as number of SNPs per ROH frequency class of 1% (e.g. from 0 to 1%). The last class includes all SNPs with a ROH frequency above 35%. [file 12711_2020_583_MOESM1_ESM.docx]

# Additional file 1


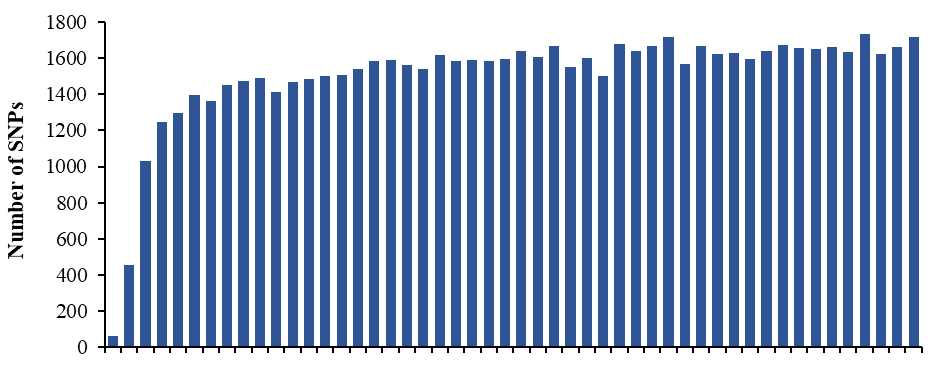


5

10

15

20

25

30

35

40

45

50

0

**Minor allele frequency (%)**

**Figure S1.** Allele frequency distribution of SNPs, shown as number of SNPs per minor allele frequency (MAF) class of 1% (e.g. from 0 to 1%).


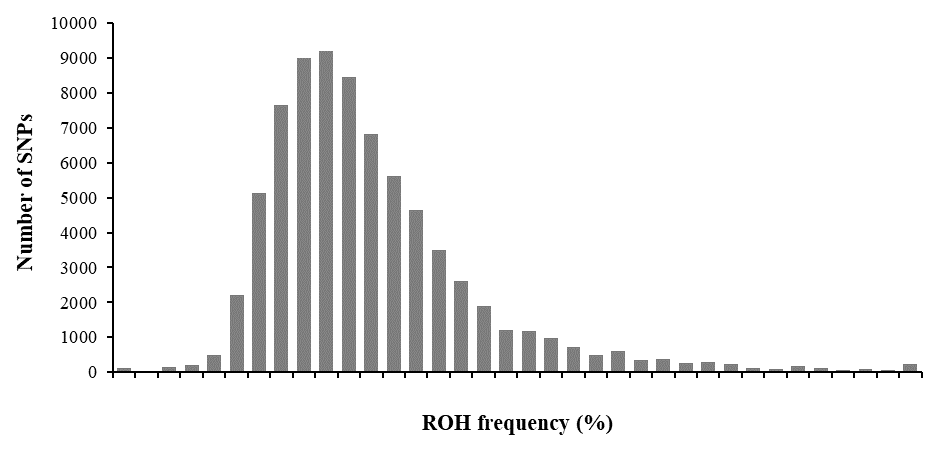


5

10

15

20

25

30

35

0

**Figure S2.** ROH frequency distribution of SNPs, shown as number of SNPs per ROH frequency class of 1% (e.g. from 0 to 1%). The last class includes all SNPs with a ROH frequency above 35%.
