# Supplementary material for: Inbreeding depression across the genome of Dutch Holstein Friesian dairy cattle
Source: Genet Sel Evol. 2020 Oct 28;52:64. doi: 10.1186/s12711-020-00583-1 (PMC7594306; doi:10.1186/s12711-020-00583-1)
Supplement: Supplementary file 2 — Additional file 2: Table S1. Construction of the R matrix for the ADR-model: SNP coding for ROH status and ROH-frequency per SNP and example of genotypes and ROH-status for 30 SNPs and two animals (j and k) in Table S1. Table S2. Construction of the R matrix for the ADR-model: example of calculating the ROH-based relationship between two animals (j and k) based on their ROH-status (${x}_{ij}$xij and ${x}_{ik}$xik) for the 30 SNPs in Table S1. Applying Eq. (S1), the ROH-based relationship between j and k is 3.0471/3.4829 = 0.8749. Note that the frequency of each SNP being in a ROH (${p}_{i}^{*}$pi∗) was assumed to be known. [file 12711_2020_583_MOESM2_ESM.docx]

**Additional file 2**

In this supplementary file, it is explained how we obtained the R matrix for the ADR-model.

**SNP coding for ROH status and ROH-frequency per SNP**

First, for each individual, we identified ROHs following the approach described in the main text (“Identification of ROH”). Then, we assigned a ROH status (denoted by $x$) of 1 to all SNPs that were in some ROH and 0 otherwise. This resulted in a vector of 0s and 1s for each individual, as illustrated in Table S1 for an example of two animals and 30 SNPs.

|  | Animal *j* | | |  | Animal *k* | | |
| --- | --- | --- | --- | --- | --- | --- | --- |
| SNP (*i*) | Allele 1 | Allele 2 | ROH-status ($x_{ij}$) |  | Allele 1 | Allele 2 | ROH-status ($x_{ik}$) |
| SNP1 | A | A | 0 |  | C | A | 0 |
| SNP2 | G | T | 0 |  | T | G | 0 |
| SNP3 | C | C | 1 |  | C | T | 0 |
| SNP4 | G | G | 1 |  | A | G | 0 |
| SNP5 | A | A | 1 |  | A | A | 0 |
| SNP6 | T | T | 1 |  | C | C | 0 |
| SNP7 | T | T | 1 |  | G | T | 0 |
| SNP8 | C | C | 1 |  | T | T | 0 |
| SNP9 | A | A | 1 |  | G | A | 0 |
| SNP10 | G | G | 1 |  | G | G | 0 |
| SNP11 | T | T | 1 |  | T | A | 0 |
| SNP12 | C | C | 1 |  | C | T | 0 |
| SNP13 | A | A | 1 |  | A | A | 1 |
| SNP14 | G | G | 1 |  | G | G | 1 |
| SNP15 | T | T | 1 |  | T | T | 1 |
| SNP16 | C | C | 1 |  | C | C | 1 |
| SNP17 | T | T | 1 |  | T | T | 1 |
| SNP18 | G | G | 1 |  | G | G | 1 |
| SNP19 | G | G | 1 |  | G | G | 1 |
| SNP20 | T | T | 1 |  | T | T | 1 |
| SNP21 | T | A | 0 |  | A | A | 1 |
| SNP22 | A | C | 0 |  | C | C | 1 |
| SNP23 | T | C | 0 |  | C | C | 1 |
| SNP24 | A | A | 0 |  | A | A | 1 |
| SNP25 | T | C | 0 |  | C | C | 1 |
| SNP26 | T | T | 0 |  | T | T | 1 |
| SNP27 | A | T | 0 |  | T | T | 1 |
| SNP28 | C | G | 0 |  | C | G | 0 |
| SNP29 | G | G | 0 |  | G | A | 0 |
| SNP30 | A | T | 0 |  | T | T | 0 |

**Table S1** Example of genotypes and ROH-status for 30 SNPs and two animals (*j* and *k*).

Based on the 0/1 ROH-status of all individuals, it was determined how often each SNP was in a ROH. For the *i^th^* SNP, this frequency was denoted by $p_{i}^{*}$. The distribution of $p_{i}^{*}$ across the genome is shown in Figure 1C of the main text.

**Calculating the ROH-based relationship**

The ROH-based relationship matrix (**R**) was built following similar reasoning as that of VanRaden method 1 [25] for computing a genomic relationship matrix. The **R** was defined as:

| $\boldsymbol{R}=\frac{\sum_{i} \left( x_{ij}-p_{i}^{*} \right)\left( x_{ik}-p_{i}^{*} \right)}{\sum_{i} p_{i}^{*}q_{i}^{*}}$ | (S1) |
| --- | --- |

where $x_{ij}$ is the ROH-status (coded as 0/1) of animal $j$ at the $i^{th}$ SNP, $x_{ik}$ is the ROH-status of animal $k$ at the $i^{th}$ SNP, $p_{i}^{*}$ is the frequency of SNP $i$ being in a ROH, and $q_{i}^{*}$ is the frequency of SNP $i$ not being in a ROH. In this notation, the numerator represents the covariance between the ROH-status of individuals, which is $\left( x_{j}-E[x] \right)*\left( x_{k}-E\left[ x \right] \right)$ for a single SNP, and the denominator represents the variance, which is $p(1-p)$ for a binary variable. In Table S2, it is shown how the ROH-based relationship between the two individuals from Table S1 can be calculated. Note that the $p_{i}^{*}$-values in this example were assumed to be known (otherwise, if $p_{i}^{*}$was based on only the two individuals, it would always have been 0, 0.5, or 1).

| SNP *i* | $x_{ij}$ | $x_{ik}$ | $p_{i}^{*}$ | $\left( x_{ij}-p_{i}^{*} \right)\left( x_{ik}-p_{i}^{*} \right)$ | $p_{i}^{*}q_{i}^{*}$ |
| --- | --- | --- | --- | --- | --- |
| SNP1 | 0 | 0 | 0.03 | 0.0009 | 0.0291 |
| SNP2 | 0 | 0 | 0.05 | 0.0025 | 0.0475 |
| SNP3 | 1 | 0 | 0.05 | -0.0475 | 0.0475 |
| SNP4 | 1 | 0 | 0.10 | -0.09 | 0.09 |
| SNP5 | 1 | 0 | 0.10 | -0.09 | 0.09 |
| SNP6 | 1 | 0 | 0.10 | -0.09 | 0.09 |
| SNP7 | 1 | 0 | 0.10 | -0.09 | 0.09 |
| SNP8 | 1 | 0 | 0.12 | -0.1056 | 0.1056 |
| SNP9 | 1 | 0 | 0.12 | -0.1056 | 0.1056 |
| SNP10 | 1 | 0 | 0.12 | -0.1056 | 0.1056 |
| SNP11 | 1 | 0 | 0.14 | -0.1204 | 0.1204 |
| SNP12 | 1 | 0 | 0.16 | -0.1344 | 0.1344 |
| SNP13 | 1 | 1 | 0.18 | 0.6724 | 0.1476 |
| SNP14 | 1 | 1 | 0.18 | 0.6724 | 0.1476 |
| SNP15 | 1 | 1 | 0.20 | 0.64 | 0.16 |
| SNP16 | 1 | 1 | 0.20 | 0.64 | 0.16 |
| SNP17 | 1 | 1 | 0.22 | 0.6084 | 0.1716 |
| SNP18 | 1 | 1 | 0.25 | 0.5625 | 0.1875 |
| SNP19 | 1 | 1 | 0.25 | 0.5625 | 0.1875 |
| SNP20 | 1 | 1 | 0.25 | 0.5625 | 0.1875 |
| SNP21 | 0 | 1 | 0.21 | -0.1659 | 0.1659 |
| SNP22 | 0 | 1 | 0.19 | -0.1539 | 0.1539 |
| SNP23 | 0 | 1 | 0.19 | -0.1539 | 0.1539 |
| SNP24 | 0 | 1 | 0.15 | -0.1275 | 0.1275 |
| SNP25 | 0 | 1 | 0.13 | -0.1131 | 0.1131 |
| SNP26 | 0 | 1 | 0.13 | -0.1131 | 0.1131 |
| SNP27 | 0 | 1 | 0.09 | -0.0819 | 0.0819 |
| SNP28 | 0 | 0 | 0.07 | 0.0049 | 0.0651 |
| SNP29 | 0 | 0 | 0.07 | 0.0049 | 0.0651 |
| SNP30 | 0 | 0 | 0.04 | 0.0016 | 0.0384 |
|  |  |  | **SUM:** | 3.0471 | 3.4829 |

**Table S2** Example of calculating ROH-based relationship between two animals (*j* and *k*) based on their ROH-status ($x_{ij}$ and $x_{ik}$) for 30 SNPs. Applying equation (S1), the ROH-based relationship between *j* and *k* is 3.0471/3.4829 = 0.8749. Note that the frequency of each SNP being in a ROH ($p_{i}^{*}$) was assumed to be known.

**Computation of R in practice**

In practice, to obtain the **R** matrix, we first converted the 0/1 ROH-status to 0/2 values. We then applied VanRaden’s formula [25] on these 0/2 values in *calc_grm* [24]. The formula of VanRaden [25] is:

$$\boldsymbol{G}=\frac{\sum_{i} \left( y_{ij}-2p_{i} \right)*\left( y_{ik}-{2p}_{i} \right)}{\sum_{i} 2p_{i}q_{i}}$$

where $y$ and $p$ are SNP allele counts and frequencies, respectively. In our case, the $y$-counts actually equalled $2x$ (where $x$ was the 0/1 ROH-status). For a single SNP, replacing $y$ by $2x$ in the formula results in a numerator of:

$$\left( 2x_{j}-2p \right)*\left( 2x_{k}-2p \right)=4x_{j}x_{k}-4px_{j}-4px_{k}-4p^{2}$$

instead of the intended numerator of:

$\left( x_{j}-p \right)*\left( x_{k}-p \right)=x_{j}x_{k}-px_{j}-px_{k}-p^{2}$.

Thus, the numerator was a factor 4 too big. At the same time, the denominator was a factor 2 too big, namely $2pq$ instead of $pq$. Consequently, the estimated ROH relationships were a factor 2 too big. To account for the difference in scale, we divided the obtained relationships by a factor 2. As expected, the average diagonal of the **R** after this correction was equal to 1.
