# Supplementary material for: Inbreeding depression across the genome of Dutch Holstein Friesian dairy cattle
Source: Genet Sel Evol. 2020 Oct 28;52:64. doi: 10.1186/s12711-020-00583-1 (PMC7594306; doi:10.1186/s12711-020-00583-1)

**Additional file 3**


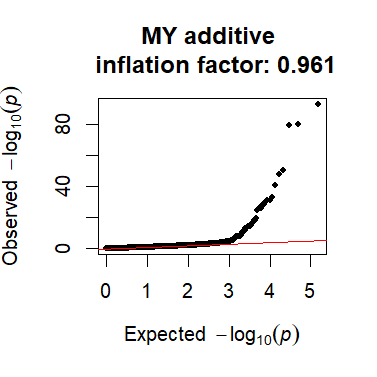

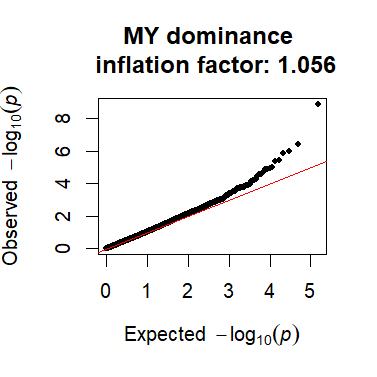

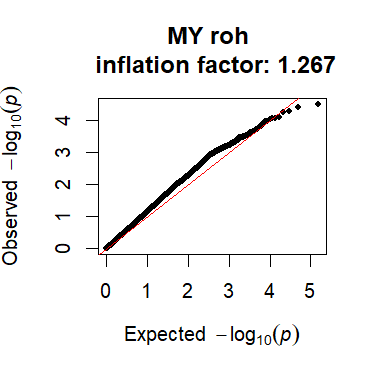

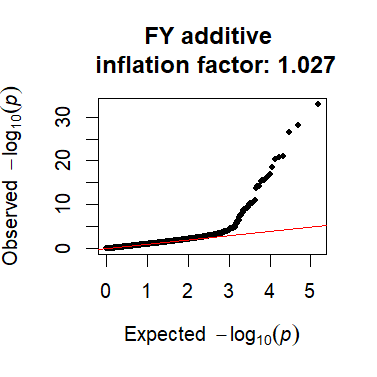

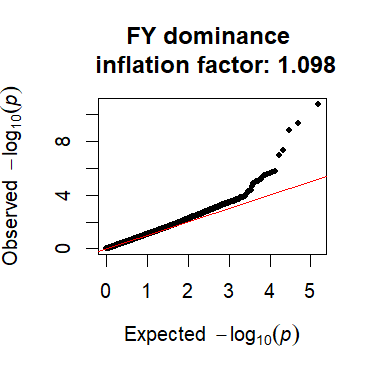

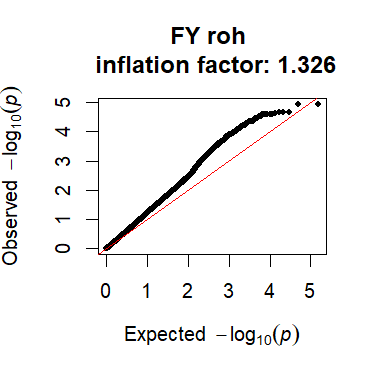

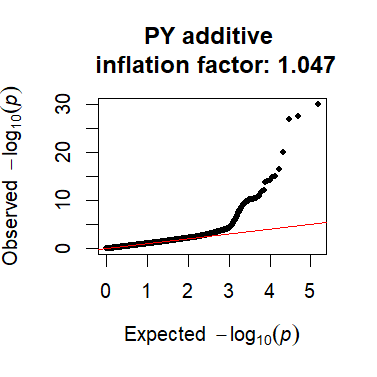

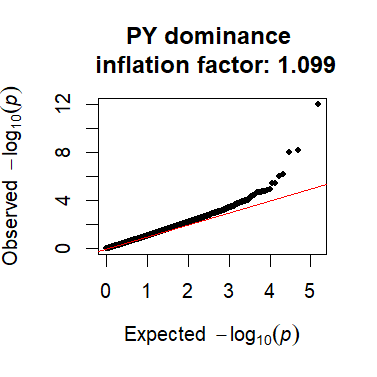

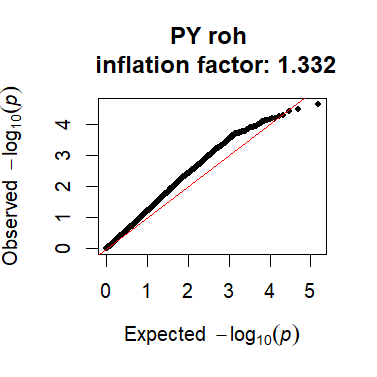

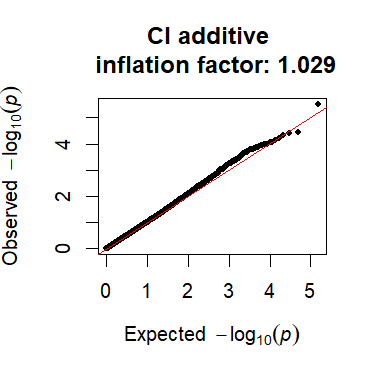

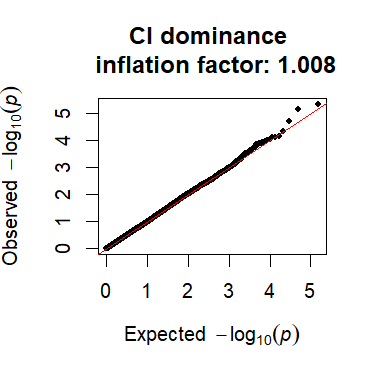

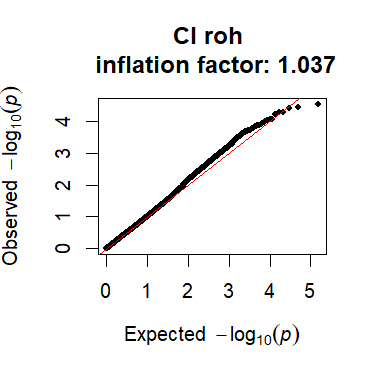


**Figure S3.** QQ-plots and genomic inflation factors for P-values corresponding to additive, dominance and ROH effects estimated by a single SNP GWAS for nine different traits (*continued on next page*). MY: 305-day milk yield; FY: 305-day fat yield; PY: 305-day protein yield; CI: calving interval; ICF: interval calving to first insemination; IFL: interval first to last insemination; CR: conception rate; SCS150 somatic cell score day 5 to 150; SCS400: somatic cell score day 151 to 400.

**Figure S3.** (continued)


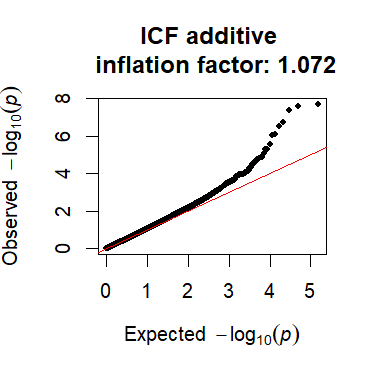

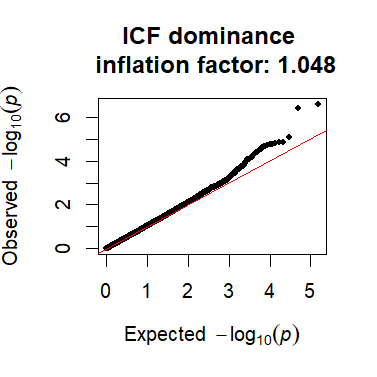

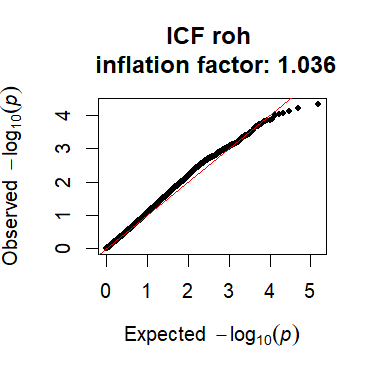

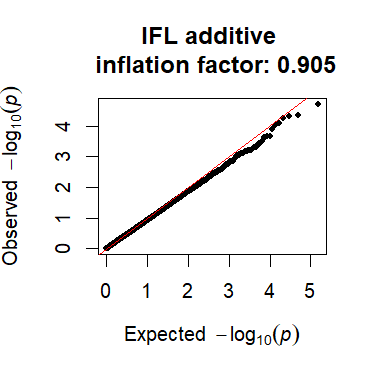

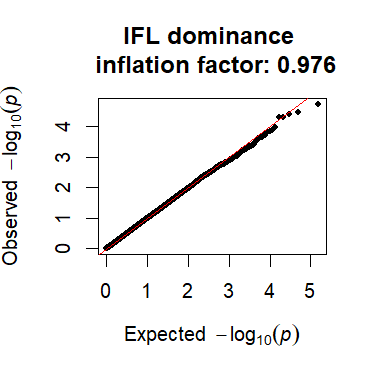

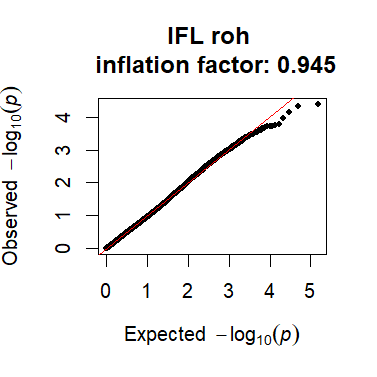

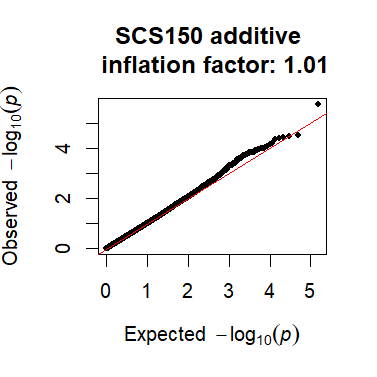

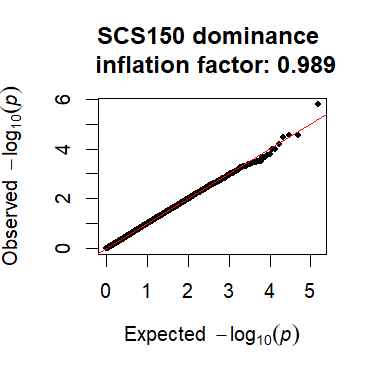

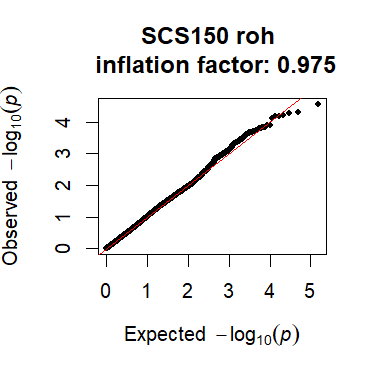

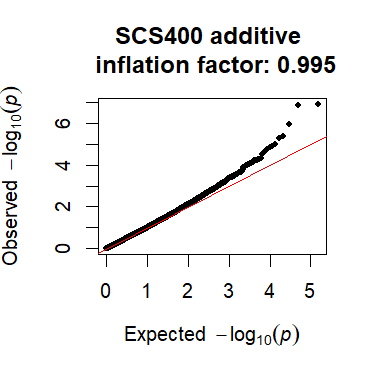

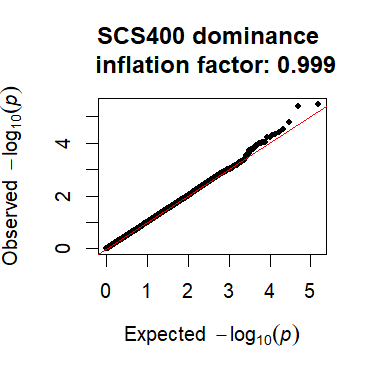

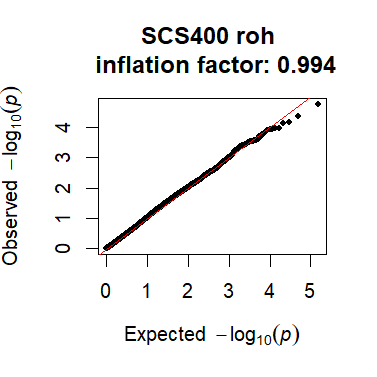

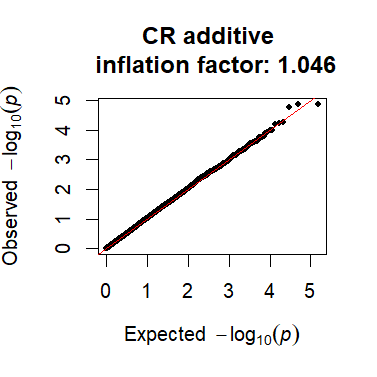

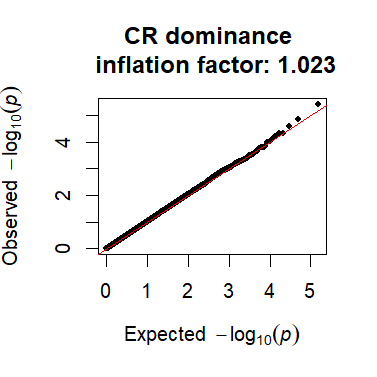

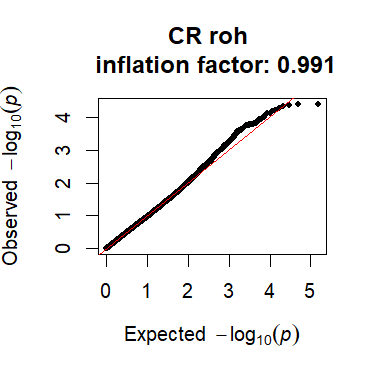

Supplement: Supplementary file 3 — Additional file 3: Figure S3. QQ-plots and genomic inflation factors for P-values corresponding to additive, dominance and ROH effects estimated by a single SNP GWAS for nine traits. MY: 305-day milk yield; FY: 305-day fat yield; PY: 305-day protein yield; CI: calving interval; ICF: interval calving to first insemination; IFL: interval first to last insemination; CR: conception rate; SCS150 somatic cell score day 5 to 150; SCS400: somatic cell score day 151 to 400. [file 12711_2020_583_MOESM3_ESM.docx]
