# Supplementary material for: Inbreeding depression across the genome of Dutch Holstein Friesian dairy cattle
Source: Genet Sel Evol. 2020 Oct 28;52:64. doi: 10.1186/s12711-020-00583-1 (PMC7594306; doi:10.1186/s12711-020-00583-1)
Supplement: Supplementary file 4 — Additional file 4: Figure S4. Additive, dominance and ROH effects for fertility and udder health traits, estimated by GREML model ADR with back-solving. CI: calving interval (d); ICF: interval calving to first insemination (d); IFL: interval first to last insemination (d); CR: conception rate (%); SCS150 somatic cell score day 5 to 150 (units); SCS400: somatic cell score day 151 to 400 (units). Effects were multiplied by 100 and divided by the genetic standard deviation (${\sigma }_{g}$σg) of the corresponding trait. Note that dominance effects for CI and ROH effects for IFL, CR, and SCS400 are not shown, because the corresponding variances were fixed to zero in the GREML (Table 2 of main text). [file 12711_2020_583_MOESM4_ESM.docx]

**Additional file 4**


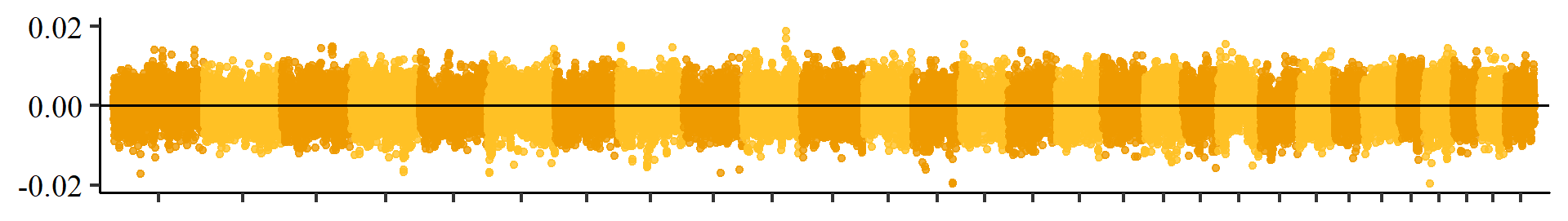

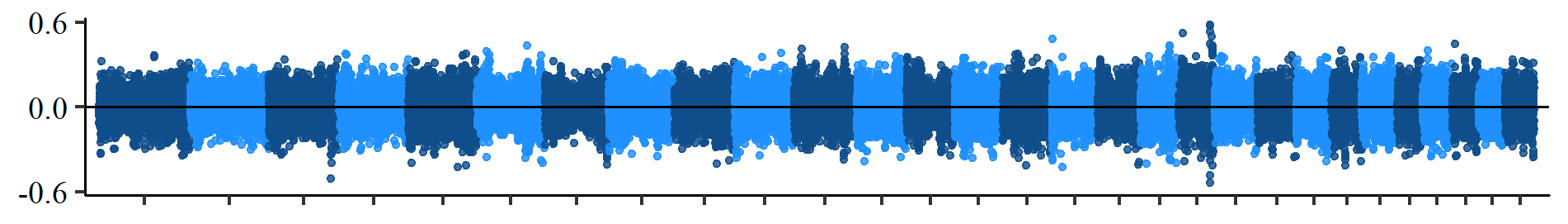

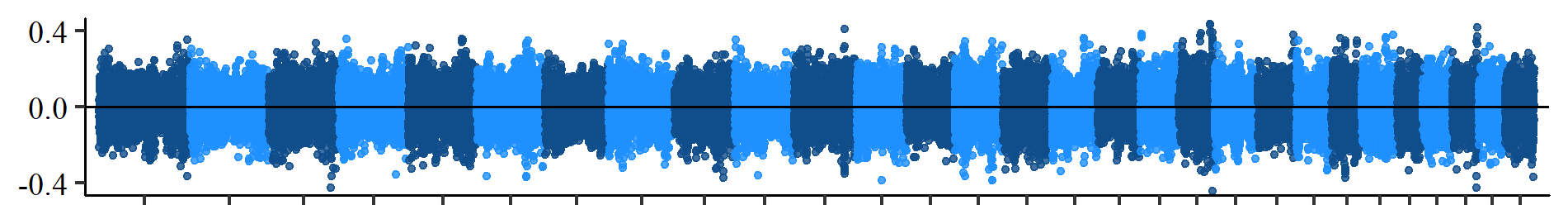

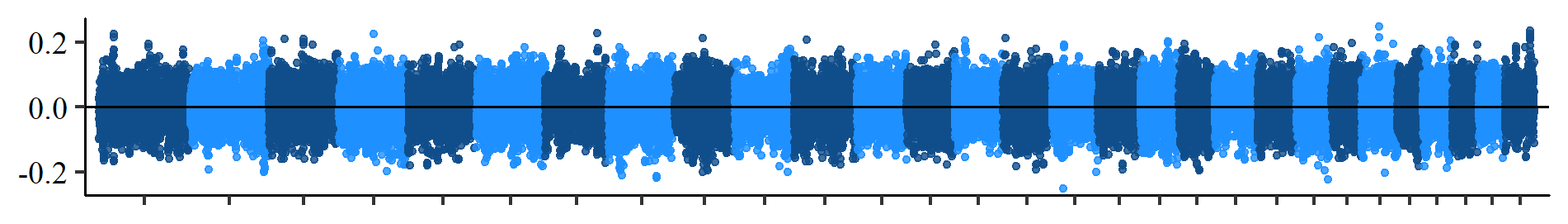

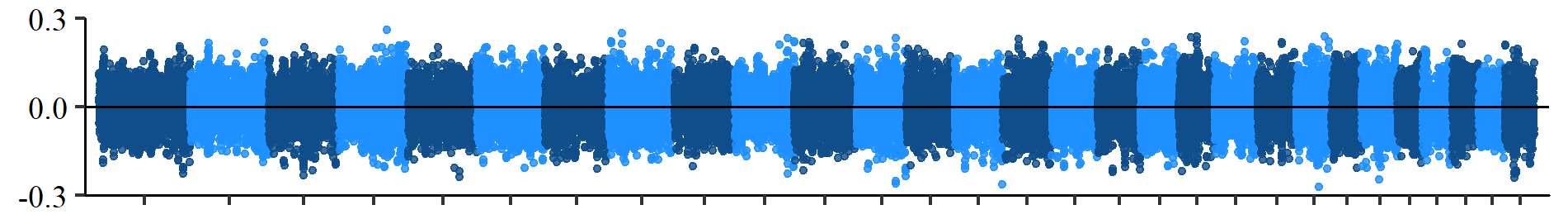

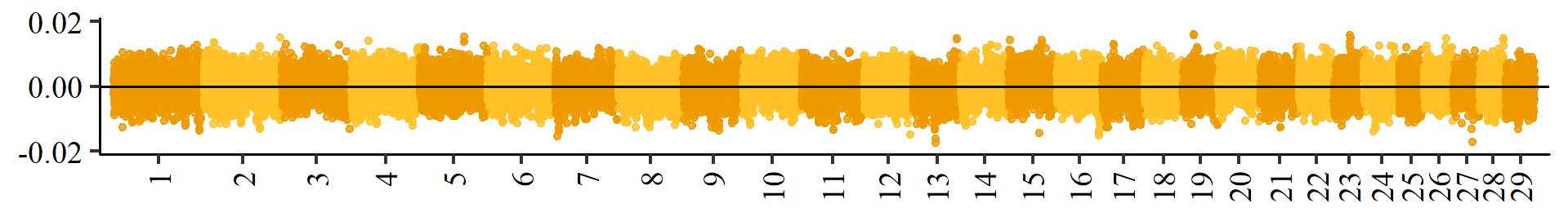

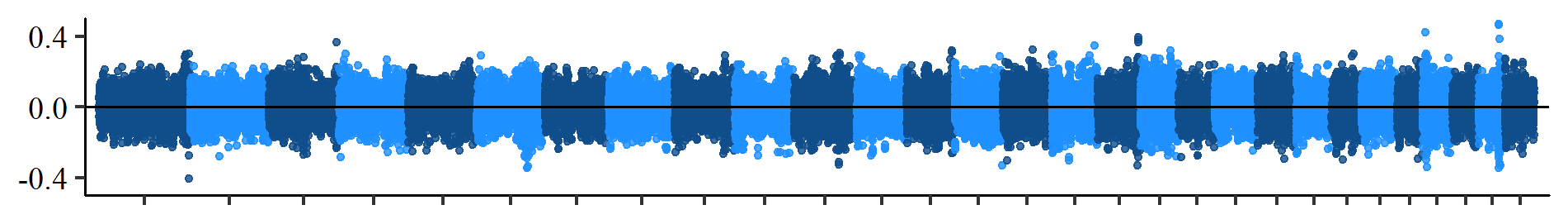

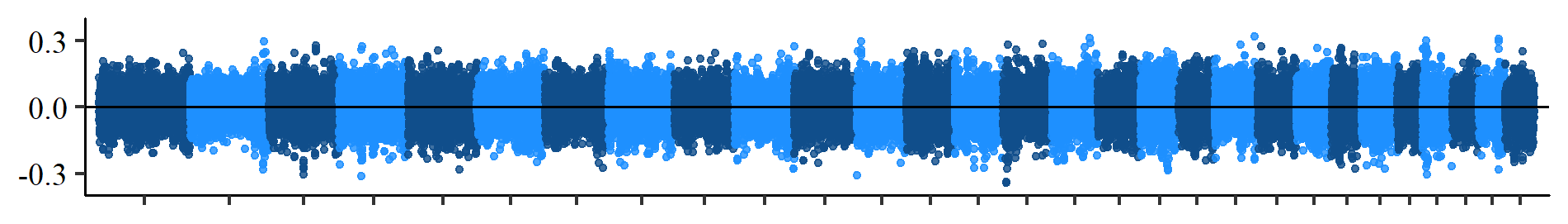


**CI: additive**

**ICF: additive**

**IFL: additive**

**CR: additive**

**SCS150: additive**

**Position (per chromosome)**

**Estimated SNP effect * 100 /** $\boldsymbol{\sigma}_{\boldsymbol{g}}$

**SCS400: additive**

**ICF: dominance**

**IFL: dominance**

**Figure S4.** Additive, dominance and ROH effects for fertility and udder health traits, estimated by GREML model ADR with backsolving (*continued on next page*). CI: calving interval (d); ICF: interval calving to first insemination (d); IFL: interval first to last insemination (d); CR: conception rate (%); SCS150 somatic cell score day 5 to 150 (units); SCS400: somatic cell score day 151 to 400 (units). Effects were multiplied by 100 and divided by the genetic standard deviation ($\sigma_{g}$) of the corresponding trait. Note that dominance effects for CI and ROH effects for IFL, CR, and SCS400 are not shown, because the corresponding variances were fixed to zero in the GREML (Table 2 of main text).

**Figure S4.** (continued)


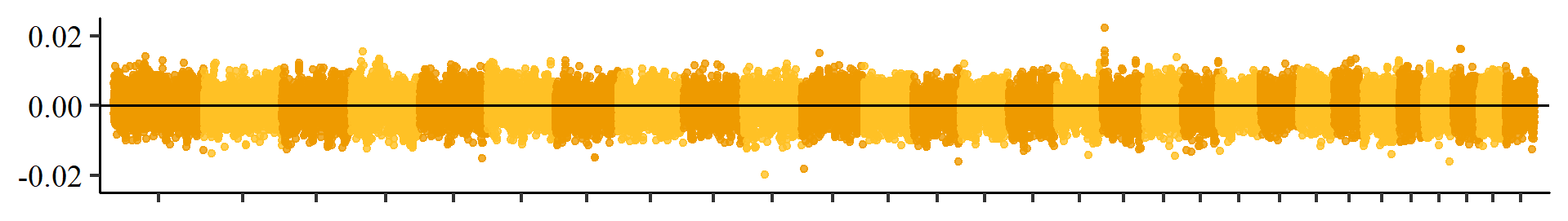

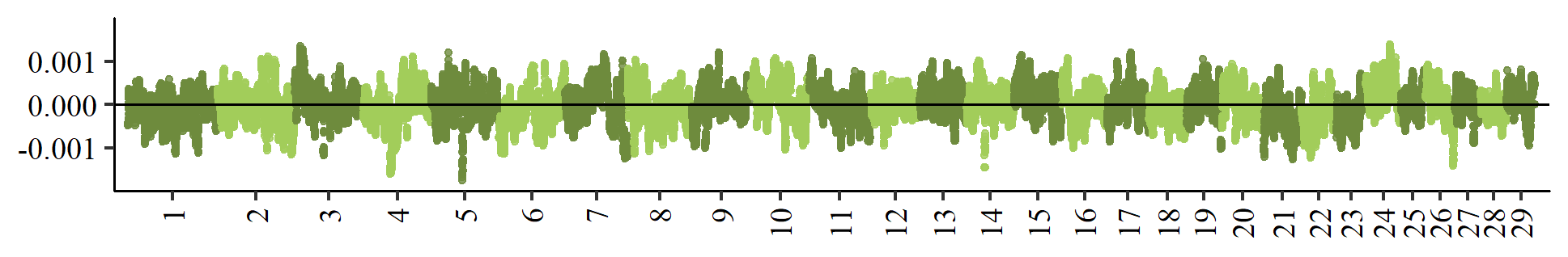

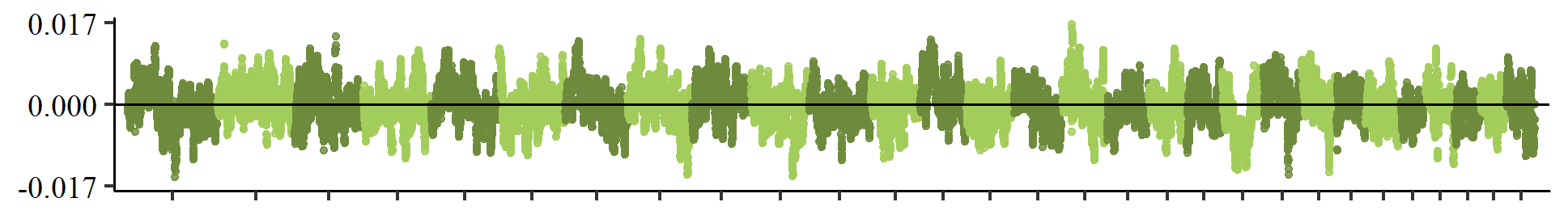

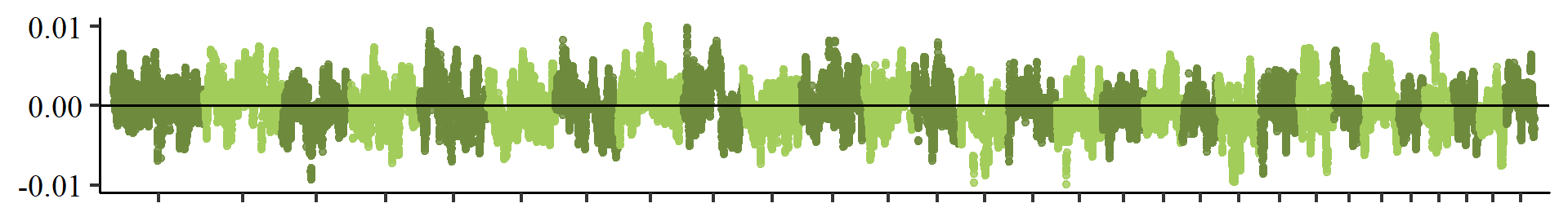

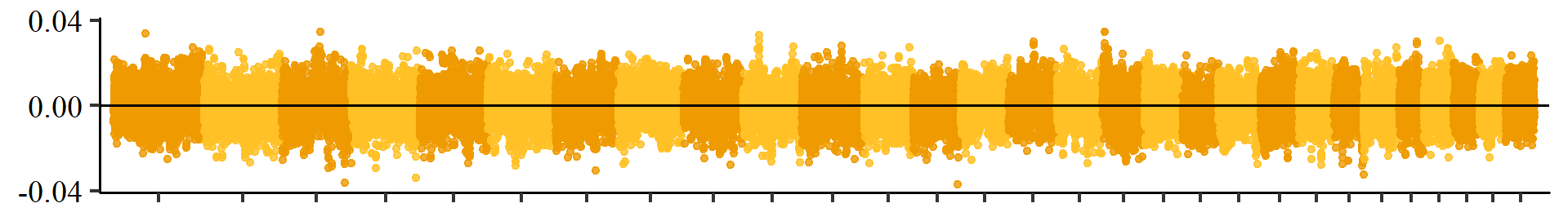

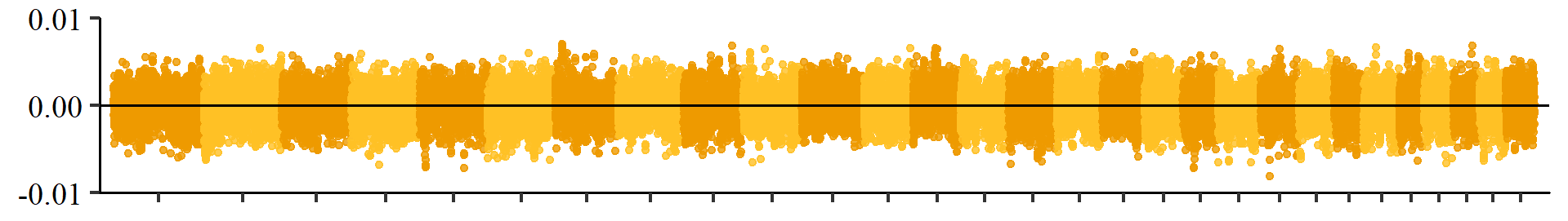


**CR: dominance**

**SCS150: dominance**

**SCS400: dominance**

**Estimated SNP effect * 100 /** $\boldsymbol{\sigma}_{\boldsymbol{g}}$

**CI: ROH**

**ICF: ROH**

**SCS150: ROH**

**Position (per chromosome)**
