# Supplementary material for: Inbreeding depression across the genome of Dutch Holstein Friesian dairy cattle
Source: Genet Sel Evol. 2020 Oct 28;52:64. doi: 10.1186/s12711-020-00583-1 (PMC7594306; doi:10.1186/s12711-020-00583-1)
Supplement: Supplementary file 5 — Additional file 5: Figure S5. Statistical significance of additive, dominance and ROH effects for fertility and udder health traits based on single SNP GWAS (continued on next page). The horizontal red line is a threshold based on 10% false-discovery rate (absence of this line indicates that all effects were below the threshold). CI: calving interval (d); ICF: interval calving to first insemination (d); IFL: interval first to last insemination (d); CR: conception rate (%); SCS150 somatic cell score day 5 to 150 (units); SCS400: somatic cell score day 151 to 400 (units). [file 12711_2020_583_MOESM5_ESM.docx]

# Additional file 5


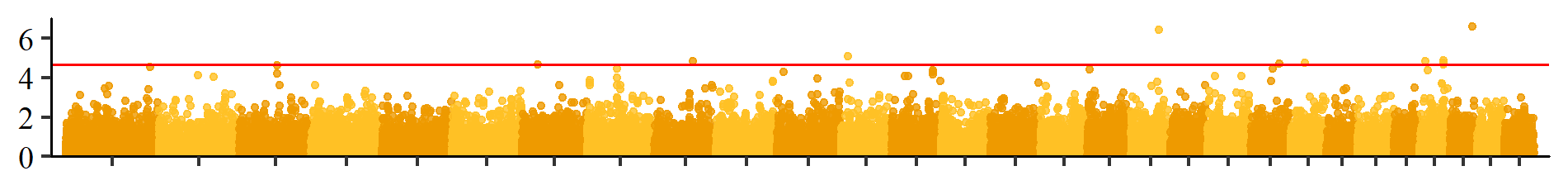

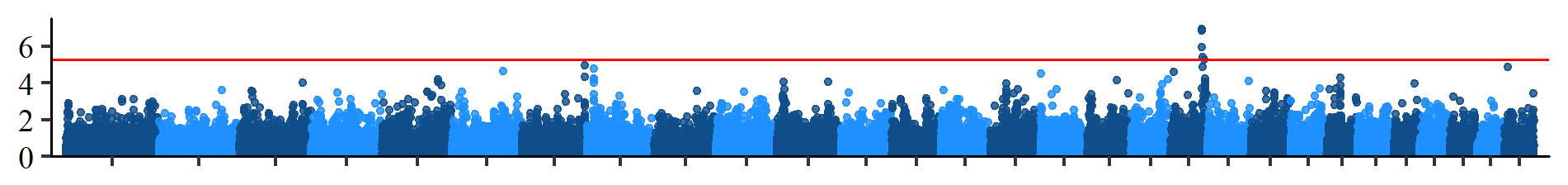

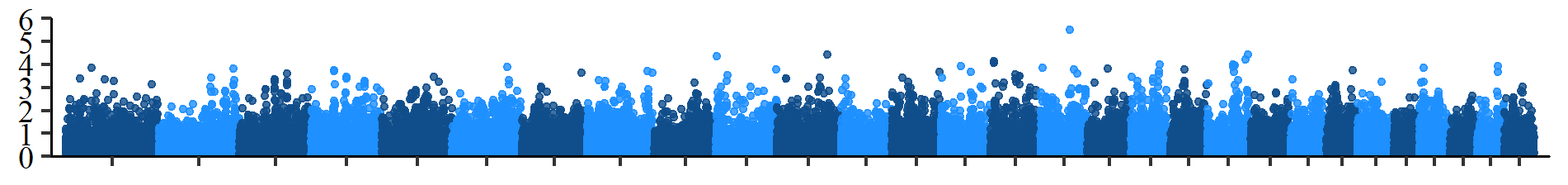

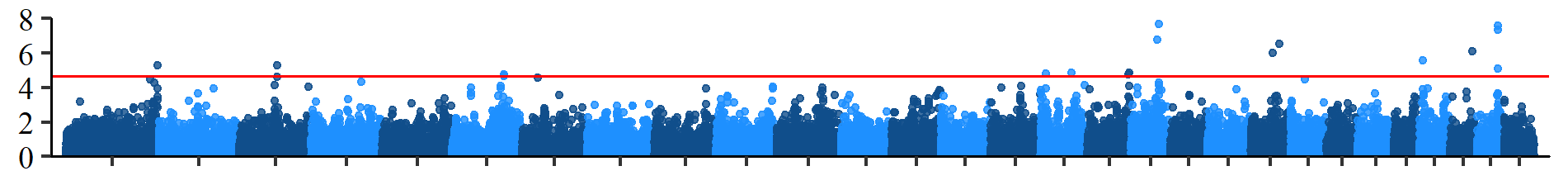

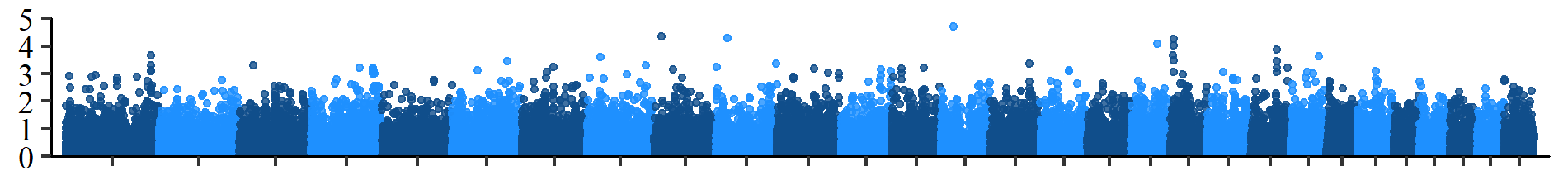

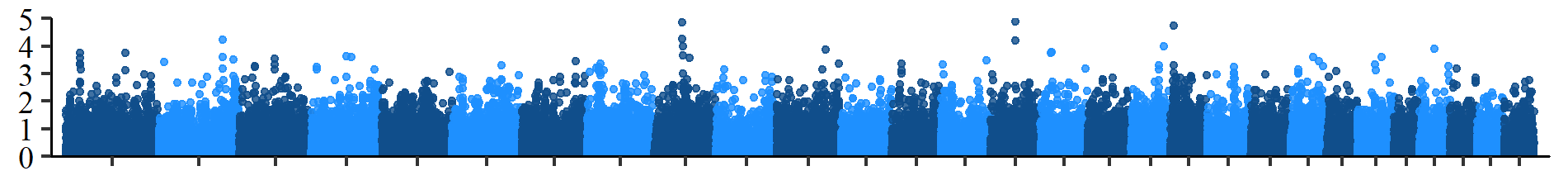

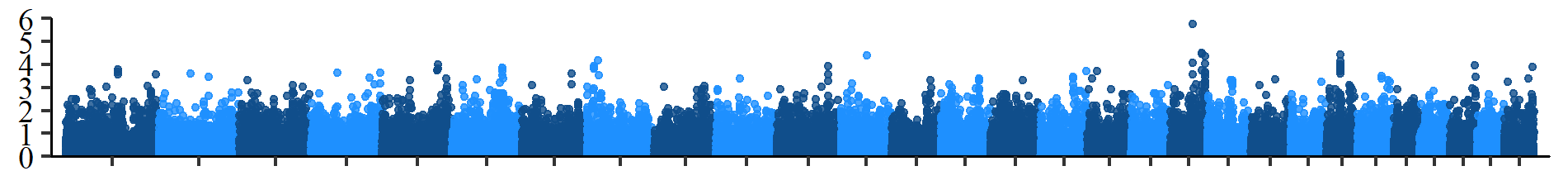

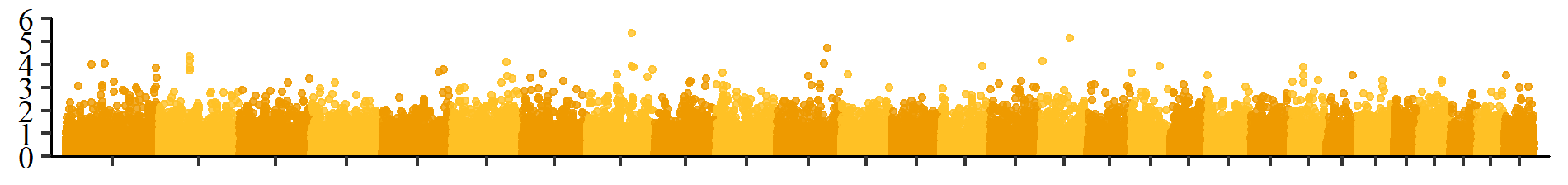

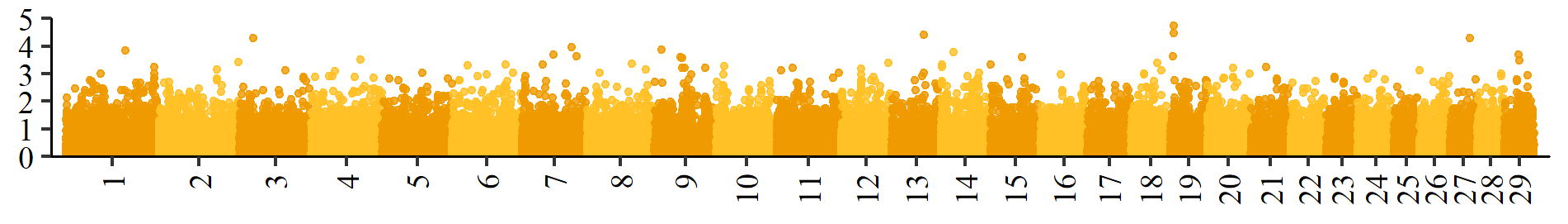


**CI: additive**

**ICF: additive**

**IFL: additive**

**CR: additive**

**SCS150: additive**

**SCS400: additive**

**ICF: dominance**

**CI: dominance**

**IFL: dominance**

**-log_10_ (*P-*value)**

**Position (per chromosome)**

**Figure S5.** Statistical significance of additive, dominance and ROH effects for fertility and udder health traits based on single SNP GWAS (*continued on next page*). The horizontal red line is a threshold based on 10% false-discovery rate (absence of this line indicates that all effects were below the threshold). CI: calving interval (d); ICF: interval calving to first insemination (d); IFL: interval first to last insemination (d); CR: conception rate (%); SCS150 somatic cell score day 5 to 150 (units); SCS400: somatic cell score day 151 to 400 (units).

**Figure S5.** (continued)


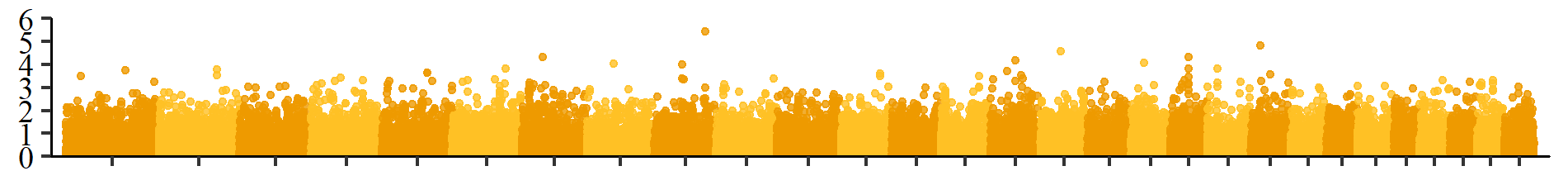

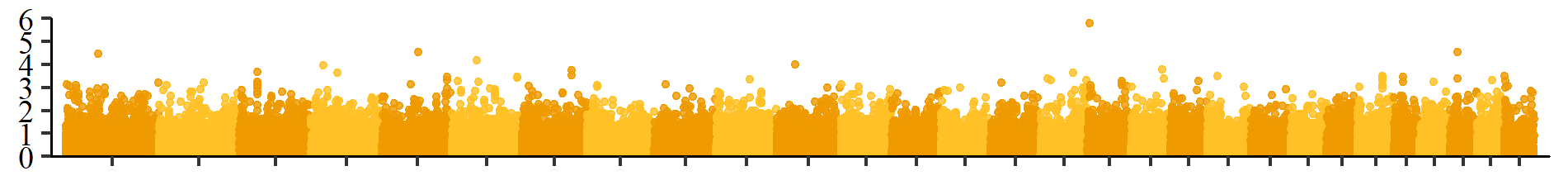

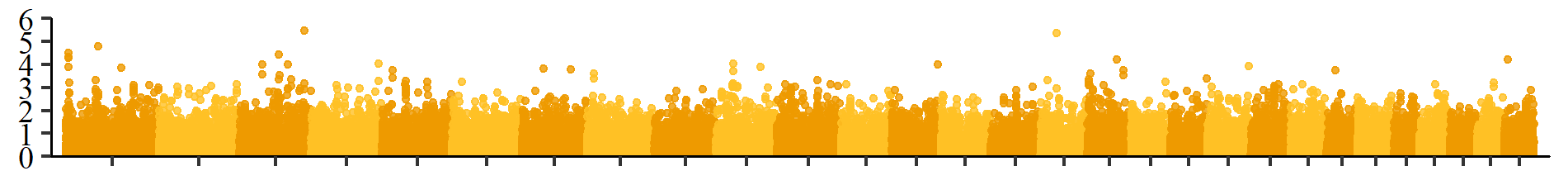

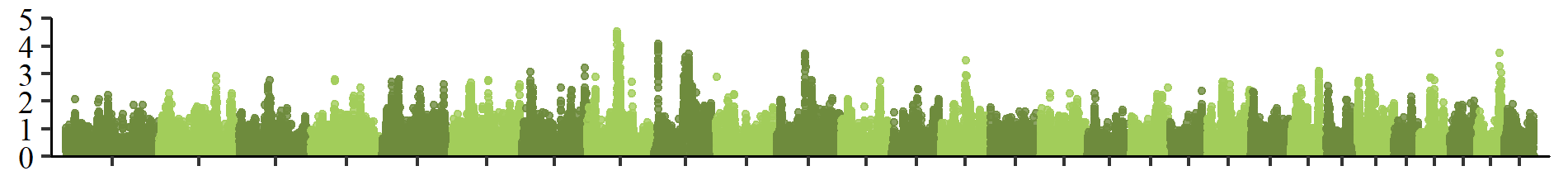

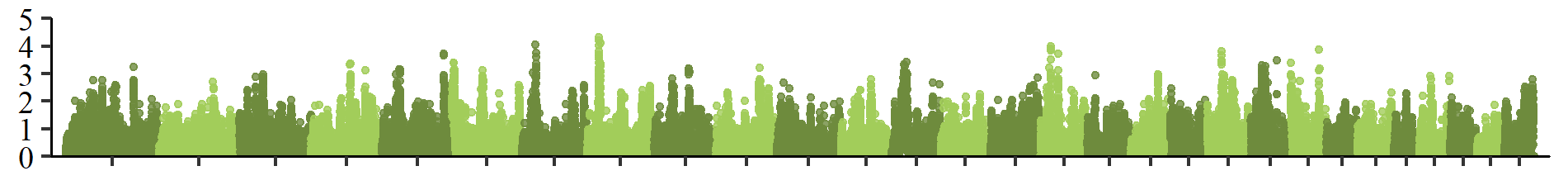

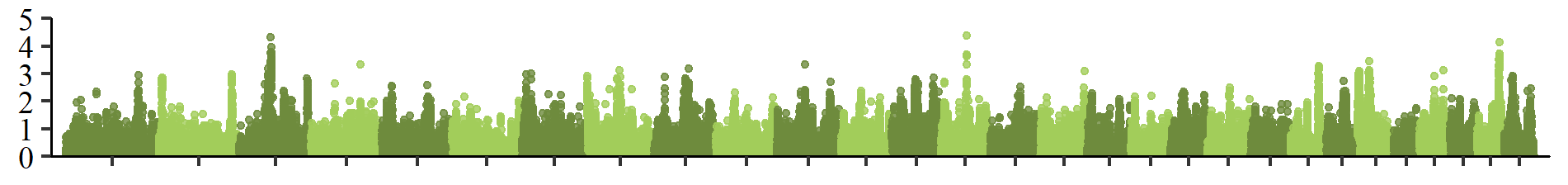

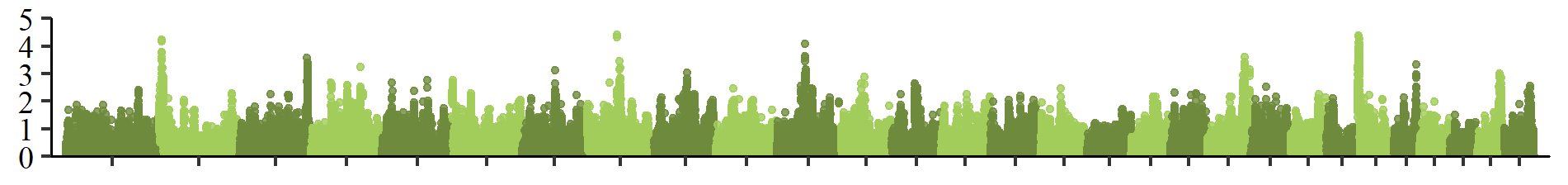

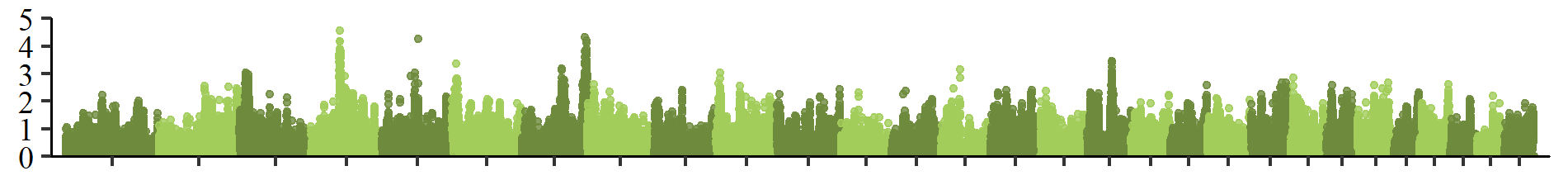

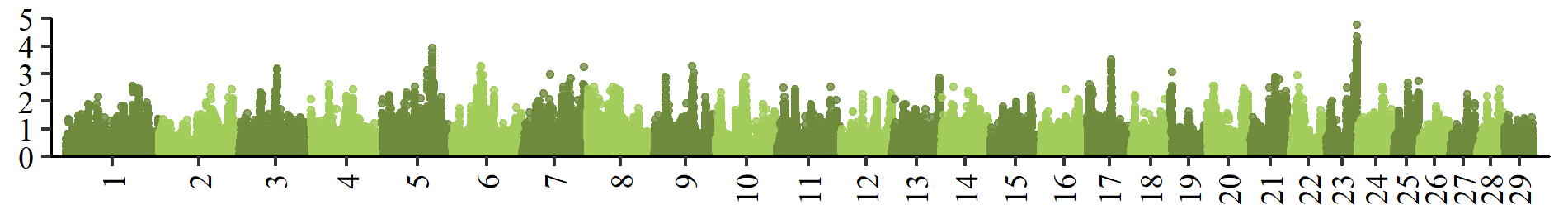


**CR: dominance**

**SCS150: dominance**

**SCS400: dominance**

**CI: ROH**

**ICF: ROH**

**IFL: ROH**

**CR: ROH**

**SCS150: ROH**

**SCS400: ROH**

**-log_10_ (*P-*value)**

**Position (per chromosome)**
