# Supplementary material for: Inbreeding depression across the genome of Dutch Holstein Friesian dairy cattle
Source: Genet Sel Evol. 2020 Oct 28;52:64. doi: 10.1186/s12711-020-00583-1 (PMC7594306; doi:10.1186/s12711-020-00583-1)
Supplement: Supplementary file 6 — Additional file 6: Figure S6. Dominance effects for yield traits, estimated by GREML (model ADR) with back-solving, for a region on chromosome 5 from 10 to 45 Mb. MY: 305-day milk yield (kg); FY: 305-day fat yield (kg); PY: 305-day protein yield (kg). Effects were multiplied by 100 and divided by the genetic standard deviation (${\sigma }_{g}$σg) of the corresponding trait. Figure S7. Statistical significance of dominance effects for yield traits, estimated by single SNP GWAS, for a region on chromosome 5 from 10 to 45 Mb. MY: 305-day milk yield. [file 12711_2020_583_MOESM6_ESM.docx]

# Additional file 6


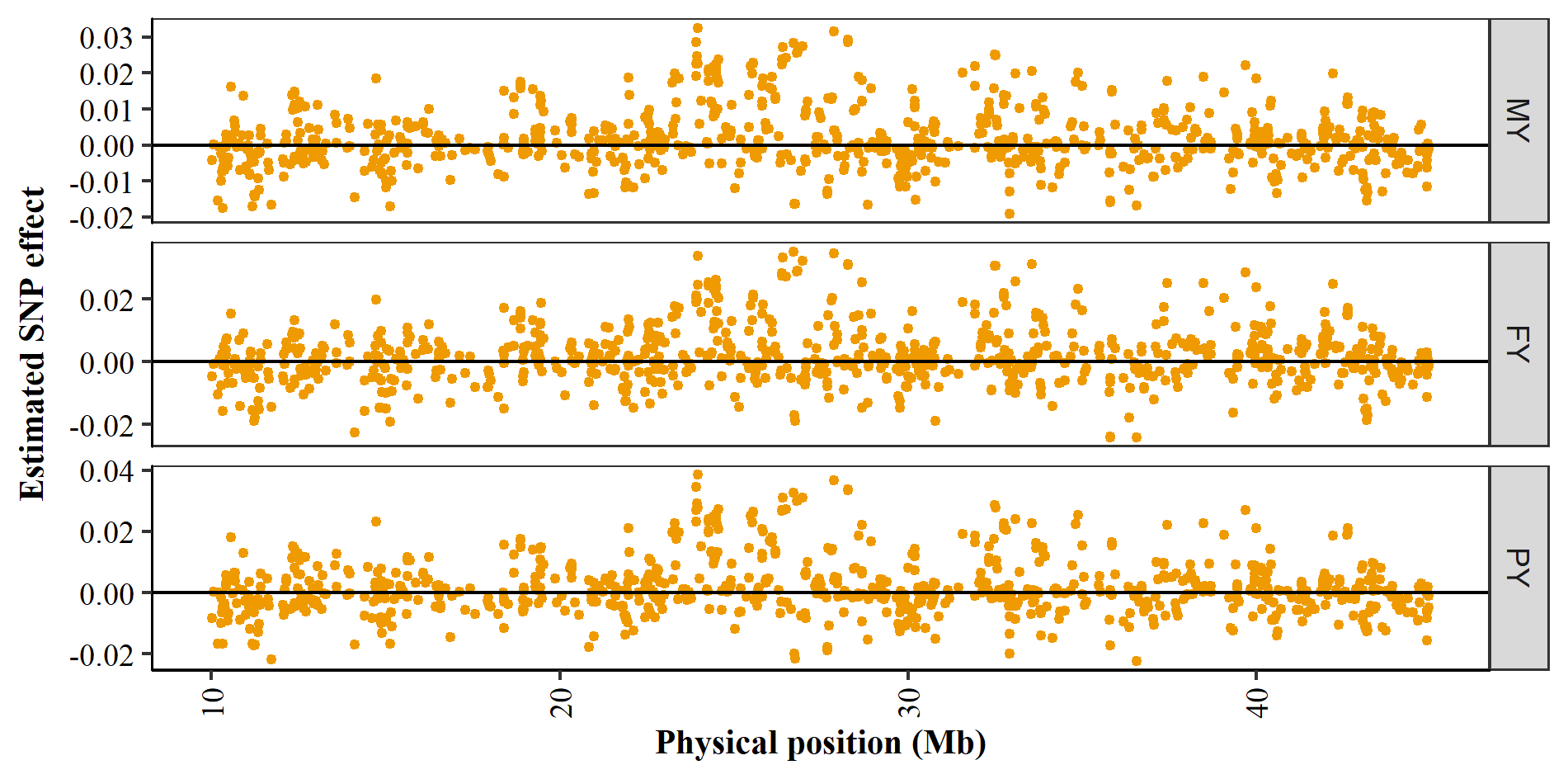


**Estimated SNP effect * 100 /** $\boldsymbol{\sigma}_{\boldsymbol{g}}$


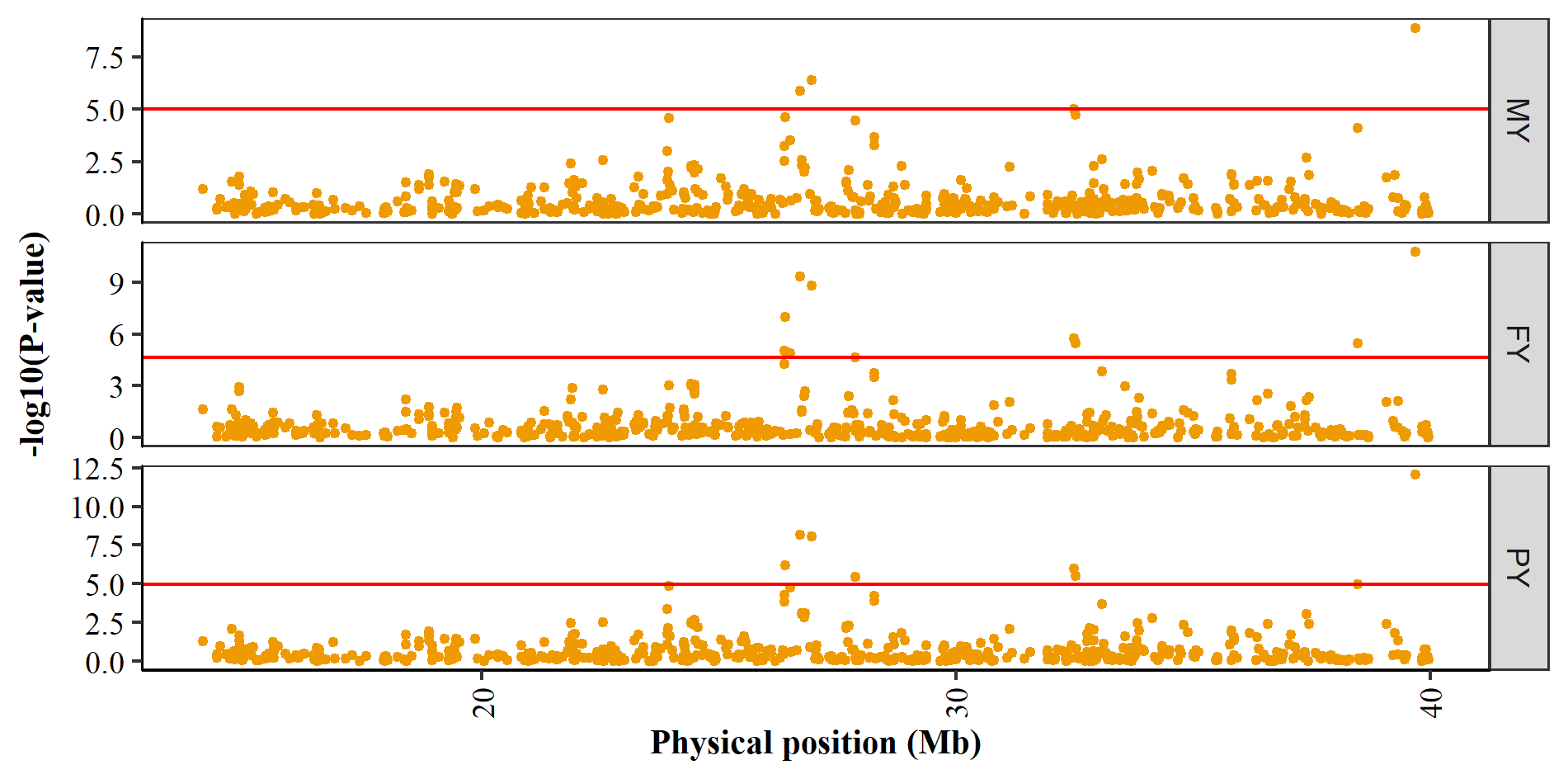
**Figure S6.** Dominance effects for yield traits, estimated by GREML (model ADR) with backsolving, for a region on chromosome 5 from 10 to 45 Mb. MY: 305-day milk yield (kg); FY: 305-day fat yield (kg); PY: 305-day protein yield (kg). Effects were multiplied by 100 and divided by the genetic standard deviation ($\sigma_{g}$) of the corresponding trait.

**Figure S7.** Statistical significance of dominance effects for yield traits, estimated by single SNP GWAS, for a region on chromosome 5 from 10 to 45 Mb. MY: 305-day milk yield; FY: 305-day fat yield; PY: 305-day protein yield.
