# Supplementary material for: Inbreeding depression across the genome of Dutch Holstein Friesian dairy cattle
Source: Genet Sel Evol. 2020 Oct 28;52:64. doi: 10.1186/s12711-020-00583-1 (PMC7594306; doi:10.1186/s12711-020-00583-1)
Supplement: Supplementary file 7 — Additional file 7: Table S3. Estimated variance components1 for additive + ROH model (AR) and nine traits2, with standard errors in parentheses. Table S4. Comparison of goodness of fit of different GREML models for nine traits. [file 12711_2020_583_MOESM7_ESM.docx]

**Additional file 7**

|  |  | Trait | | | | | | | | |
| --- | --- | --- | --- | --- | --- | --- | --- | --- | --- | --- |
| Model | Parameter | MY | FY | PY | CI | ICF | IFL | CR | SCS150 | SCS400 |
| AR | $\sigma_{P}^{2}$ | 1356096 | 1812.80 | 1268.73 | 4215.08 | 717.600 | 3056.2 | 12.6991 | 17693.3 | 16336.5 |
|  | $\sigma_{A}^{2}/\sigma_{P}^{2}$ (%) | 41.10 (0.81) | 33.28 (0.82) | 30.90 (0.81) | 5.01 (0.43) | 6.37 (0.49) | 3.13 (0.35) | 2.35 (0.32) | 9.25 (0.54) | 11.94 (0.61) |
|  | $\sigma_{R}^{2}/\sigma_{P}^{2}$ (%) | 0.24 (0.10) | 0.33 (0.11) | 0.31 (0.12) | 0.07 (0.12) | 0.15 (0.13) | 0* | 0* | 0.03 (0.11) | 0.05 (0.11) |
|  | $\sigma_{A}^{2}/\sigma_{G}^{2}$ (%) | 99.42 (0.24) | 99.03 (0.34) | 99.01 (0.37) | 98.53 (2.25) | 97.69 (1.95) | 100* | 100* | 99.63 (1.14) | 99.61 (0.90) |
|  | $\sigma_{R}^{2}/\sigma_{G}^{2}$ (%) | 0.58 (0.24) | 0.97 (0.34) | 0.99 (0.37) | 1.47 (2.25) | 2.31 (1.95) | 0* | 0* | 0.37 (1.14) | 0.39 (0.90) |

**Table S3.** Estimated variance components^1^ for additive + ROH model (AR) and nine traits^2^, with standard errors in parentheses.

*The ROH variance component was fixed to 0 (because its initial estimate was slightly negative)

^1^$\sigma_{P}^{2}$: phenotypic variance (excluding the herd-year-season variance); $\sigma_{A}^{2}$: additive genetic variance; $\sigma_{ROH}^{2}$: ROH variance; $\sigma_{G}^{2}$: genetic variance ($\sigma_{A}^{2}+\sigma_{R}^{2}$).

^2^MY: 305-day milk yield (kg); FY: 305-day fat yield (kg); PY: 305-day protein yield (kg); CI: calving interval (days); ICF: interval calving to first insemination (days); IFL: interval first to last insemination (days); CR: conception rate (%); SCS150 somatic cell score day 5 to 150 (1000+100*[log2 of cells/mL]); SCS400: somatic cell score day 151 to 400 (1000+100*[log2 of cells/mL]).

**Table S4.** Comparison of goodness of fit of different GREML models^1^ for nine traits^2^.

|  | Difference in maximum log-likelihood | |  | P-value | |
| --- | --- | --- | --- | --- | --- |
| Trait | AR - A | ADR - AR |  | AR vs A | ADR vs AR |
| MY | 4.253 | 1.355 |  | 0.002 | 0.050 |
| FY | 6.061 | 1.308 |  | <0.001 | 0.053 |
| PY | 5.203 | 1.058 |  | <0.001 | 0.073 |
| CI | 0.236 | 0.000 |  | 0.246 | 0.500 |
| ICF | 0.775 | 0.043 |  | 0.107 | 0.384 |
| IFL | 0* | 0.026 |  | NA* | 0.410 |
| CR | 0* | 0.004 |  | NA* | 0.465 |
| SCS150 | 0.053 | 0.060 |  | 0.372 | 0.364 |
| SCS400 | 0.099 | 0.366 |  | 0.328 | 0.196 |

*The ROH variance component was fixed to 0 (because its initial estimate was slightly negative)

^1^A: additive model; AR: additive + ROH model; ADR: additive + dominance + ROH model.

^2^MY: 305-day milk yield; FY: 305-day fat yield; PY: 305-day protein yield; CI: calving interval; ICF: interval calving to first insemination; IFL: interval first to last insemination; CR: conception rate; SCS150 somatic cell score day 5 to 150; SCS400: somatic cell score day 151 to 400.
