# Supplementary material for: Inbreeding depression across the genome of Dutch Holstein Friesian dairy cattle
Source: Genet Sel Evol. 2020 Oct 28;52:64. doi: 10.1186/s12711-020-00583-1 (PMC7594306; doi:10.1186/s12711-020-00583-1)
Supplement: Supplementary file 8 — Additional file 8: Table S5. Correlations between variance components estimates1 from the ADR model for nine traits. Correlations were calculated from the average information matrix from mtg2 output. [file 12711_2020_583_MOESM8_ESM.docx]

**Additional file 8**

**Table S5.** Correlations between variance components estimates^1^ from the ADR model for nine traits^2^. Correlations were calculated from the average information matrix from mtg2 output.

|  | Correlation between variance component estimates | | | | | | | | | |
| --- | --- | --- | --- | --- | --- | --- | --- | --- | --- | --- |
| Trait | $\sigma_{A}^{2}$ & $\sigma_{D}^{2}$ | $\sigma_{A}^{2}$ & $\sigma_{ROH}^{2}$ | $\sigma_{D}^{2}$ & $\sigma_{ROH}^{2}$ | $\sigma_{A}^{2}$ & $\sigma_{HYS}^{2}$ | $\sigma_{A}^{2}$ & $\sigma_{E}^{2}$ | $\sigma_{D}^{2}$ & $\sigma_{HYS}^{2}$ | $\sigma_{D}^{2}$ & $\sigma_{E}^{2}$ | $\sigma_{ROH}^{2}$ & $\sigma_{HYS}^{2}$ | $\sigma_{ROH}^{2}$ & $\sigma_{E}^{2}$ | $\sigma_{HYS}^{2}$ & $\sigma_{E}^{2}$ |
| MY | 0.00 | -0.01 | -0.42 | 0.00 | -0.24 | 0.00 | -0.45 | 0.00 | 0.09 | -0.15 |
| FY | 0.00 | -0.01 | -0.41 | 0.00 | -0.23 | 0.00 | -0.44 | 0.00 | 0.08 | -0.15 |
| PY | 0.00 | -0.02 | -0.41 | 0.00 | -0.23 | 0.00 | -0.45 | 0.00 | 0.09 | -0.14 |
| CI | 0* | -0.04 | 0* | 0.00 | -0.22 | 0.00 | 0* | 0.01 | -0.11 | -0.27 |
| ICF | 0.02 | -0.05 | -0.45 | -0.01 | -0.21 | -0.01 | -0.45 | 0.01 | 0.11 | -0.19 |
| IFL | -0.01 | 0* | 0* | 0.00 | -0.19 | 0.00 | -0.43 | 0.00 | 0* | -0.24 |
| CR | -0.01 | 0* | 0* | 0.00 | -0.18 | 0.00 | -0.44 | 0.00 | 0* | -0.24 |
| SCS150 | 0.02 | -0.04 | -0.48 | 0.00 | -0.21 | -0.01 | -0.42 | 0.00 | 0.11 | -0.21 |
| SCS400 | 0.00 | 0* | 0* | 0.00 | -0.22 | 0.00 | -0.43 | 0.00 | 0* | -0.22 |

*One of the variance components was fixed to 0 (because its initial estimate was slightly negative).

^1^$\sigma_{A}^{2}$: additive genetic variance; $\sigma_{D}^{2}$: dominance variance; $\sigma_{ROH}^{2}$: ROH variance; $\sigma_{HYS}^{2}$: herd-year-season variance; $\sigma_{E}^{2}$: residual variance.

^2^MY: 305-day milk yield (kg); FY: 305-day fat yield (kg); PY: 305-day protein yield (kg); CI: calving interval (days); ICF: interval calving to first insemination (days); IFL: interval first to last insemination (days); CR: conception rate (%); SCS150 somatic cell score day 5 to 150 (1000+100*[log2 of cells/mL]); SCS400: somatic cell score day 151 to 400 (1000+100*[log2 of cells/mL]).
